# Identification of circulating miRNA alterations in diabetes patients excluding periodontitis effects: insights into target gene downregulation in diabetic complications

**DOI:** 10.1080/07853890.2025.2567609

**Published:** 2025-11-10

**Authors:** Hae Seul Lee, Yeuni Yu, Hyun-Joo Kim, Jung-Min Oh, Hae-Ryoun Park, Yun Hak Kim

**Affiliations:** ^a^ Department of Anatomy, School of Medicine, Pusan National University, Yangsan, Republic of Korea; ^b^ Medical Research Institute, Pusan National University, Yangsan, Republic of Korea; ^c^ Department of Periodontology, Dental and Life Science Institute, Pusan National University, School of Dentistry, Yangsan, Republic of Korea; ^d^ Periodontal Disease Signaling Network Research Center, School of Dentistry, Pusan National University, Yangsan, Republic of Korea; ^e^ Department of Periodontics and Dental Research Institute, Pusan National University Dental Hospital, Yangsan, Republic of Korea; ^f^ Department of Oral Biochemistry, School of Dentistry, Pusan National University, Yangsan, Republic of Korea; ^g^ Institute for Future Earth, Pusan National University, Busan, Republic of Korea; ^h^ Department of Oral Pathology, Dental & Life Science Institute, School of Dentistry, Pusan National University, Yangsan, Republic of Korea; ^i^ Department of Periodontology and Dental Research Institute, Pusan National University Dental Hospital, Yangsan, Republic of Korea; ^j^ Department of Biomedical Informatics, School of Medicine, Pusan National University, Yangsan, Republic of Korea; ^k^ Research Institute for Convergence of Biomedical Science and Technology, Pusan National University Yangsan Hospital, Yangsan, Republic of Korea

**Keywords:** Diabetes mellitus, periodontitis, periodontitis with diabetes mellitus (PDDM), circulating exosomal microRNAs, miRNA biomarkers, diabetic foot ulcer, diabetic nephropathy, diabetic retinopathy, Pancreatic dysfunction

## Abstract

**Background:**

Diabetes mellitus (DM) induces systemic complications through chronic metabolic dysregulation. Circulating exosomal microRNAs (miRNAs) are emerging as key regulators of post-transcriptional gene expression and may drive diabetes-associated pathologies. Although miRNAs have been widely studied in diabetes, the characterization of PD-independent miRNA signatures across tissues remains limited. This study aimed to identify DM-specific miRNA alterations and their contribution to systemic metabolic dysfunction independent of PD.

**Methods:**

Exosomes were isolated from plasma samples, and small RNA sequencing was performed to identify differentially expressed miRNAs (DE-miRs) using the limma R package. Predicted target genes were identified using TargetScan and validated through bulk RNA sequencing datasets from four tissues—foot, kidney, pancreas, and retina. Differentially expressed genes (DEGs) were analyzed, followed by Gene Ontology Biological Process (GOBP) and Kyoto Encyclopedia of Genes and Genomes (KEGG) pathway enrichment to elucidate diabetes-related mechanisms.

**Results:**

We identified 9 upregulated and 6 downregulated DE-miRs specific to the diabetic group. TargetScan predicted 216 upregulated and 64 downregulated target genes. Functional validation revealed that these genes were enriched in pathways related to glucose metabolism, cellular stress response, and tissue repair. Notably, *SREK1* and *GLIPR1* were commonly detected across all four tissues, suggesting potential systemic regulators of diabetes-related complications.

**Conclusion:**

This study suggests that circulating exosomal miRNAs, independent of periodontitis, may function as systemic regulators in diabetes. Unlike previous studies, which did not distinguish co-morbid periodontitis, we specifically defined PD-independent miRNA signatures and validated their cross-organ regulatory effects on target genes. Our results revealed a cross-organ miRNA–mRNA regulatory network and identified common regulatory targets. These findings provide insights into both systemic and organ-specific mechanisms underlying diabetic complications and highlight the potential of miRNAs as biomarkers and therapeutic targets.

## Graphical abstract

**Figure:**
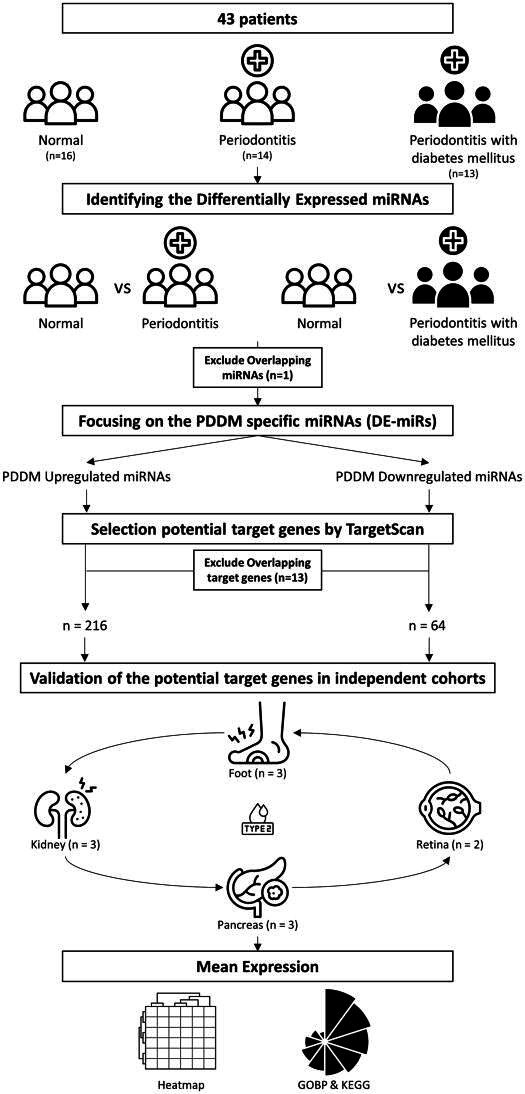

## 1. Introduction

Periodontitis (PD) is a chronic inflammatory disease causing destruction of tooth-supporting structures, including the alveolar bone and periodontal ligaments [1]. PD affects oral health locally and has systemic repercussions, particularly when accompanied by metabolic conditions, such as diabetes mellitus (DM) [2]. When PD and DM coexist, as in periodontitis with diabetes mellitus (PDDM), both conditions worsen due to shared mechanisms such as chronic inflammation, oxidative stress, and impaired immune responses [3]. Particularly, PD is a common DM complication, significantly contributing to comorbidities and obscuring DM-specific systemic effects. Distinguishing the systemic effects of DM from those of combined PDDM pathologies is essential to advance disease understanding and improve management strategies.

Type 2 diabetes mellitus (T2DM), the most prevalent form of diabetes worldwide, results from a combination of insulin resistance and altered insulin secretion [4,5]. The major risk factors for T2DM include an unhealthy diet, physical inactivity, obesity, genetic predisposition, and stress [6]. Although T2DM has traditionally been more prevalent among older adults, its incidence is rapidly rising among younger populations due to the increasing rates of obesity and adoption of Western diets [7,8]. Early diagnosis is challenging because T2DM is often asymptomatic in the initial stages, which can lead to complications such as cardiovascular disease, nephropathy, neuropathy, and retinopathy [9,10]. These complications significantly reduce patients’ quality of life and impose a substantial burden on healthcare systems [11]. Therefore, integrated treatment strategies targeting both the local and systemic effects of T2DM are critical to mitigate these effects.

Recent studies have highlighted exosomes as nano-sized extracellular vesicles that can mediate intercellular communication by transporting bioactive molecules, including proteins, lipids, and RNA [12]. These exosomes have emerged as key mediators with significant diagnostic and therapeutic potential [13]. Among these, exosomal microRNAs (miRNAs) in the blood system have attracted attention because of their accessibility and active roles in disease processes [14,15]. Circulating exosomal miRNAs regulate gene expression by suppressing target gene translation and modulating key biological processes such as inflammation, tissue repair, and immune responses [16].

Given that both PD and DM are systemic diseases with widespread effects beyond their local manifestations, distinguishing patients with DM alone from those affected by both conditions remains a significant challenge [17]. To address potential confounding from PD, the study design included strict selection criteria and additional validation using independent datasets from diabetes-related tissues. Herein, we aimed to explore circulating exosomal miRNA signatures specific to diabetes mellitus, independent of periodontitis-related influences. We further sought to identify the target gene networks regulated by these miRNAs and to examine their potential involvement in systemic metabolic dysregulation and organ-specific complications associated with diabetes.

## 2. Methods

### 2.1. Subject recruitment

Healthy individuals and patients were recruited from the Department of Oral and Maxillofacial Surgery and Periodontics, Pusan National University Dental Hospital (Republic of Korea), respectively. Subjects were selected based on medical history from questionnaires and interviews; disease status was further verified by periodontal examination and HbA1c measurement. Healthy controls showed no signs of clinical inflammation, such as redness, swelling, or bleeding on probing, and had a pocket depth under 3 mm as well. Patients were selected with stage III periodontitis based on the 2017 World Workshop criteria, and HbA1c levels ranged from 6.61% to 10%. Subjects with systemic diseases other than diabetes or who had received periodontal therapy within 6 months were excluded. All subjects provided written informed consent prior to enrollment. The study was conducted between September 4, 2020 and August 31, 2021, following approval by the Institutional Review Board of Pusan National University Dental Hospital (IRB No. PNUDH-2020-032).

### 2.2. Sample collection and exosome purification

Venous blood was collected into sterile EDTA tubes. Samples were centrifuged at 2,000 g for 10 min, and plasma was separated and stored at −80 °C until use. Exosomes were isolated by precipitating exosomes within the plasma using Exo2DTM for RNA assay (EXOSOMEplus, Suwon, Republic of Korea). Additional characterization of exosomes, such as size profiling or surface marker analysis, was not performed. Therefore, the term “exosomal RNA” is used based on the isolation protocol, in alignment with common practice in extracellular RNA research, and with reference to the MISEV2018 guidelines [18].

### 2.3. RNA extraction and RNA sequencing

Exosomal RNA was extracted from human plasma using the miRNeasy Serum/Plasma Kit (Qiagen, Cat No. 217184) according to the manufacturer’s instructions. Given the fragmented nature of exosomal RNA, RNA integrity number (RIN) was not applicable. Instead, RNA quality and size distribution were assessed using the Agilent 2100 Bioanalyzer with RNA Pico and Small RNA chips. RNA concentration was quantified using the Quant-iT^™^ RiboGreen RNA Assay Kit (Thermo Fisher Scientific).

Libraries were prepared using the SMARTer smRNA-Seq Kit for Illumina (Takara Bio, Shiga, Japan), and average insert sizes ranged from 208 to 237 bp. Sequencing was conducted at Macrogen (Seoul, Republic of Korea) on an Illumina HiSeq 2500 platform using 51 base pair (bp) single-end reads with standard Illumina SBS (Sequencing By Synthesis) chemistry, generating approximately 2.0–2.5 GB of raw data per sample. Sequencing reads were aligned to the human reference genome (GRCh38), and mature miRNAs were annotated using miRBase version 22.1 and RNAcentral version 14.0.

### 2.4. Differentially expressed-miRs (DE-miRs) analysis and identification of target genes

Reads per million (RPM; small RNA counts/total counts × 1 million) were used for normalization. The normalized expression data from small RNA sequencing have been deposited in the NCBI Gene Expression Omnibus (GEO) under accession number GSE301956. DE-miRs were identified using the limma R package (version 3.60.6) [19], with thresholds of *p* < 0.05 and |log₂ fold change| > 2. Significant DE-miRs were independently filtered from comparisons between control vs. PD and control vs. PDDM. To focus on PDDM-specific miRNAs, overlapping DE-miRs between the two comparisons were excluded. Target genes of DE-miRs were predicted using TargetScan (https://www.targetscan.org/vert_80/) [20]. By intersecting their predicted target sets, 229 target genes of the upregulated miRNAs and 77 target genes of the downregulated miRNAs were identified. To reduce redundancy and clarify biological roles, 13 overlapping genes between the two sets were excluded. This refinement resulted in 216 unique target genes for the upregulated miRNAs and 64 for the downregulated miRNAs, enabling a more precise interpretation of their regulatory effects.

### 2.5. Validation datasets and differentially expressed genes (DEG) analysis

To validate systemic and organ-specific miRNA target genes, GEO datasets were selected based on data quality, sample size, and annotation completeness. Priority was given to tissues clinically relevant to diabetic complications, including the foot, kidney, pancreas, and retina. Only datasets with sufficient quality and metadata were included. Batch correction was not required, as each dataset originated from an independent study cohort and was analyzed separately by tissue type without merging, which inherently minimized batch effects and platform-related variability.

The following datasets were used: GSE68183, GSE80178, and GSE199939 for foot; GSE142025, GSE162830, and GSE163603 for kidney; GSE20966, GSE25724, and GSE164416 for pancreas; and GSE102485 and GSE160306 for retina.

DEGs were identified exclusively on the predicted target genes of DE-miRs. As this study aimed to identify potential regulatory targets in an exploratory context, a relaxed significance threshold of *p* < 0.1 was applied using Student’s t-test. In addition, mean expression differences between groups were assessed. Only genes that met both the statistical significance (*p* < 0.1) and showed a notable mean expression difference were considered significantly differentially expressed.

### 2.6. Cell culture, transfection, and quantitative RT-PCR

Human embryonic kidney cells (HEK293T, CRL-3216^™^, ATCC, Manassas, VA, USA) were cultured in Dulbecco’s Modified Eagle’s Medium (DMEM; Welgene Inc., Daegu, Republic of Korea) supplemented with 10% fetal bovine serum (FBS; Hyclone, South Logan, UT, USA) at 37 °C in a humidified incubator containing 5% CO₂ and 95% O₂.

Cells were seeded in 6-well plates and transfected the following day with either hsa-miR-144-3p or hsa-miR-140-3p mimics (AccuTarget^™^ Human miRNA mimics, Bioneer, Daejeon, Republic of Korea) and negative controls (AccuTarget^™^ Human miRNA negative control, Bioneer, Daejeon, Republic of Korea) using Lipofectamine^™^ RNAiMAX reagent (Invitrogen, Karlsruhe, Germany), according to the manufacturer’s protocol. The sequences of the mimics are listed in Supplementary Table 6.

Total RNA was extracted at 24 and 48 h post-transfection using the AccuPrep^®^ Universal RNA Extraction Kit (Bioneer). For mRNA quantification, 500 ng of RNA was reverse-transcribed using the AccuPower^®^ RocketScript^™^ Master Mix (Bioneer), and quantitative PCR was performed with AccuPower^®^ 2X GreenStar^™^ qPCR Master Mix on the CFX Duet Real-Time PCR System (Bio-Rad, Hercules, CA, USA). *SREK*1 mRNA levels were quantified using gene-specific primers provided in Supplementary Table 7. All reactions were performed in duplicate for 45 cycles, and relative gene expression was calculated using the 2^−ΔΔCt method.

For mature miRNA quantification, cDNA synthesis was performed using the Mir-X^™^ miRNA First-Strand cDNA Synthesis Kit (Clontech, TaKaRa, Tokyo, Japan) in an Agilent thermal cycler under standardized conditions. qRT-PCR was then conducted using the TB Green Advantage qPCR Premix (Clontech) on a CFX96 Real-Time PCR System (Bio-Rad). According to the manufacturer’s instructions, the mature miRNA sequence was used as the forward primer, and the universal mRQ 3′ primer included in the kit was used as the reverse primer. U6 small nuclear RNA (snRNA) served as the internal control. Forward primers used for mature miRNA quantification are listed in Supplementary Table 8.

### 2.7. Statistical analysis and visualization

DEGs were identified using the limma R package (version 3.60.6), with p-values adjusted by the false discovery rate (FDR) method. For data visualization, the R packages tidyr (version 1.3.1), ggplot2 (version 3.5.1), Eulerr (version 7.1.0), and pheatmap (version 1.0.12) were used.

To assess potential confounding effects of clinical variables, including age and body mass index (BMI), on circulating DE-miRNAs, two complementary statistical approaches were employed. First, Spearman’s rank correlation coefficients were computed between DE-miRNAs and clinical indicators, with the results visualized as dot plots and correlation matrices. Second, multiple linear regression models were fitted with age and BMI as covariates to evaluate whether group-specific DE-miRNA expression differences remained significant after adjustment. For all regression analyses, p-values were corrected for multiple testing using the Benjamini–Hochberg false discovery rate (FDR) method.

Heatmaps were generated using normalized expression data scaled by the min–max method to highlight differences in selected target gene expression. To explore common features among DEGs between groups, Gene Ontology Biological Process (GOBP) and Kyoto Encyclopedia of Genes and Genomes (KEGG) pathway analyses were conducted. Only terms with p-values below 0.05 were considered statistically significant and included for further interpretation. To enhance relevance, diabetes-related terms from these analyses were selected and visualized. A term–gene network was constructed based on enriched GO terms and their corresponding target genes.

To evaluate the effect of miRNA mimics on *SREK1* expression, independent t-tests were performed at 24 and 48 h post-transfection, comparing mimic-treated groups (miR-140 or miR-144) to the control.

## 3. Results

### 3.1. Clinical characteristics and cohort descriptions

A total of 43 participants were enrolled and classified into three groups: 16 healthy controls, 14 patients with periodontitis (PD), and 13 patients with both periodontitis and diabetes mellitus (PDDM) (Table 1). The PDDM group had the highest average age (62.69 ± 9.24 years), followed by the PD group (55.36 ± 7.92 years), whereas the healthy group was comparatively younger (43.69 ± 10.93 years). Body mass index (BMI) was relatively comparable across groups. Periodontal clinical parameters such as attachment loss (AL), probing depth (PD), gingival index (GI), and erythrocyte sedimentation rate (ESR) were not significantly different between the PD and PDDM groups. However, the PDDM group exhibited increased plaque levels (PL, 52.8 ± 23.45) and recession (RE, 0.80 ± 0.80) compared to the PD group. Furthermore, inflammatory and metabolic markers—including C-reactive protein (CRP, 12.37 ± 35.95 mg/L) and glycated hemoglobin (HbA1C, 7.78 ± 1.12%)—were markedly elevated in the PDDM group, clearly distinguishing it from both the healthy and PD groups. Given the substantial age differences between groups, the potential confounding effects of age and BMI were further evaluated through correlation analysis and regression analysis, which is described in a subsequent section.

**Table 1.** Characteristics of subjects.

Categories	Subjects	Group		
		Normal	PD	PDDM
N	43	16	14	13
Age (yrs, mean ± SD)	53.23 ± 12.26	43.69 ± 10.93	55.36 ± 7.92	62.69 ± 9.24
BMI (kg/m²)	24.4 ± 3.16	22.69 ± 3.11	25.65 ± 3.36	24.38 ± 2.47
AL (mm)	3.31 ± 1.29	2.13 ± 0.12	3.92 ± 1.07	3.68 ± 1.43
PD (mm)	2.85 ± 0.91	2.09 ± 0.10	3.49 ± 1.06	2.88 ± 0.70
RE	0.44 ± 0.64	0.00 ± 0.00	0.42 ± 0.48	0.80 ± 0.80
PL (mean ± SD)	42.16 ± 21.73	21.04 ± 3.83	49.10 ± 16.19	52.8 ± 23.45
GI	0.38 ± 0.39	0.03 ± 0.04	0.68 ± 0.27	0.38 ± 0.42
ESR(pre) (mm/hr)	7.17 ± 7.05	4.75 ± 2.71	8.71 ± 9.44	7.00 ± 5.77
CRP(pre) (mg/L)	5.25 ± 22.08	0.60 ± 0.14	1.29 ± 1.11	12.37 ± 35.95
HbA1C (%)	5.94 ± 1.70	4.61 ± 1.86	5.39 ± 0.37	7.78 ± 1.12
HbA1C (mM/M)	44.74 ± 15.12	33.60 ± 3.15	35.37 ± 4.09	61.68 ± 11.30
HbA1C (mg/dL)	132.53 ± 39.69	103.29 ± 8.27	107.93 ± 10.74	177.01 ± 29.67

Abbreviations: AL, attachment loss; PD, probing depth; RE, recession; PL, plaque level; GI, gingival index; ESR(pre), erythrocyte sedimentation rate (pre-treatment); CRP(pre), C-reactive protein (pre-treatment); HbA1C, glycated hemoglobin.

### 3.2. Sequencing results of exosomal RNAs

**Figure 1.**
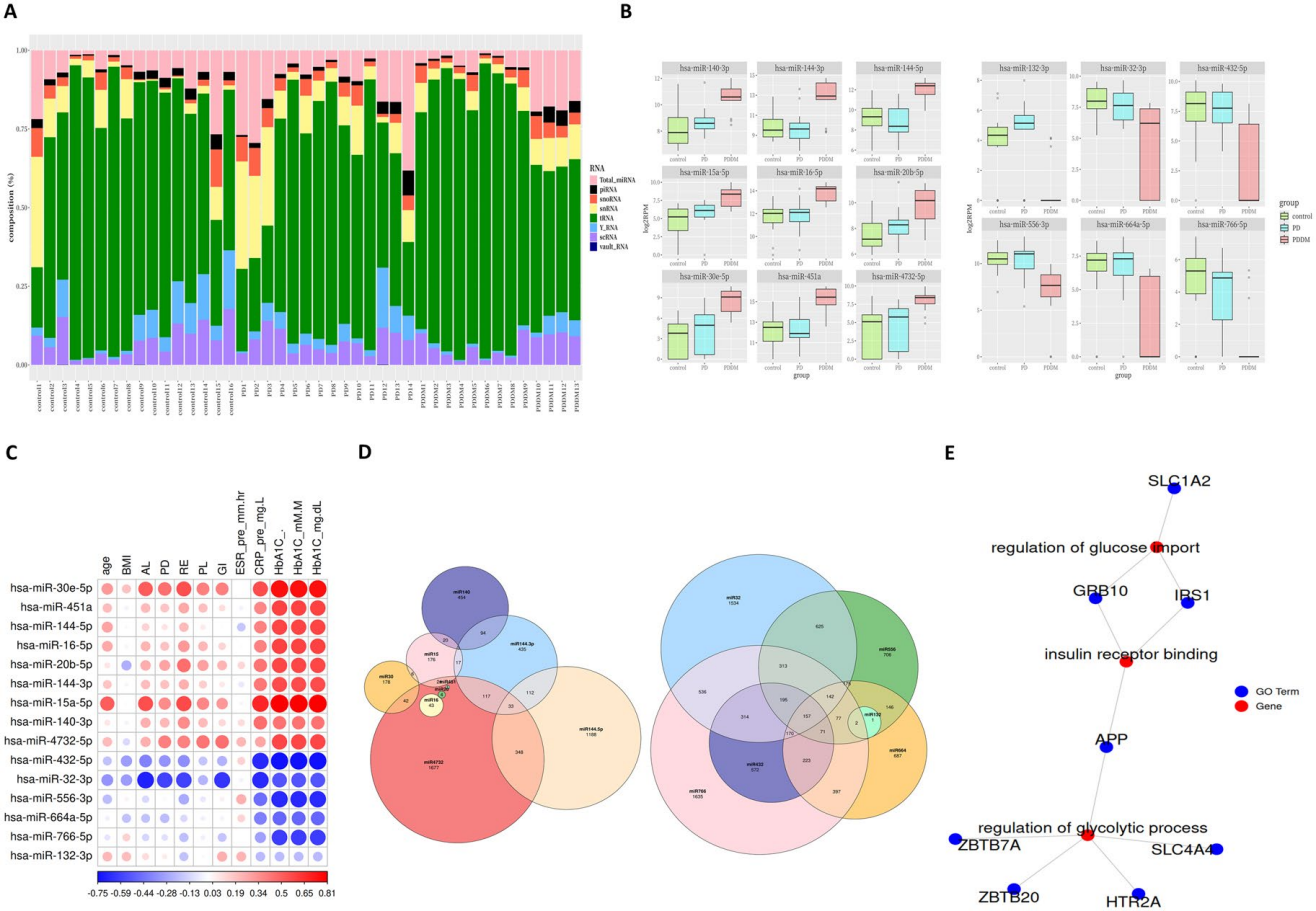
Sequencing results of circulating exosomes and comparative analysis of differential miRNA expression among control, PD, and PDDM groups. (A) Relative composition of sncRNA: a stacked bar plot representing the relative abundance of sncRNA types, including tRNAs, miRNAs, snRNAs, and others, across individual samples. (B) DE-miRs: Boxplots of upregulated (left) and downregulated (right) miRNAs in PDDM compared to controls identified using the limma package with a moderated student’s t-test (*p* < 0.05, |log₂FC| > 2). Expression values are log-transformed. (C) Correlation matrix: Spearman correlation coefficients between DE-miRs and clinical variables (age, BMI, HbA1c, CRP, AL, GI). Color intensity indicates correlation strength (red: positive, blue: negative). (D) Venn diagrams of predicted miRNA target genes: Target genes of upregulated (left) and downregulated (right) miRNAs predicted by TargetScan. (E) GO term–target gene network for upregulated miRNAs: a network diagram linking diabetes-related GO:BP terms (e.g. glucose import, insulin receptor binding, regulation of glycolytic process) to their corresponding target genes downregulated at the mRNA level.

Exosomes contain not only miRNAs, but also a diverse range of small noncoding RNAs (sncRNAs) [12]. To assess differences in sncRNA composition across groups and identify RNA types predominant under specific physiological or pathological conditions, the relative abundance of exosomal RNAs was quantified for each sample (Figure 1A). Among the eight identified sncRNA types, transfer RNAs (tRNAs) were the most abundant across nearly all samples, regardless of group. This was followed by total miRNAs and small nuclear RNAs (snRNAs). Notably, tRNAs consistently dominated in the control, PD, and PDDM groups, suggesting a shared core RNA profile in circulating exosomes.

### 3.3. PDDM-specific circulating DE-miRs and their target genes

DE-miRs were identified by comparing control vs. PD and control vs. PDDM groups (Table 2). To focus on diabetes-specific changes, overlapping miRNAs (e.g. hsa-miR-363-3p) were excluded, resulting in 9 upregulated and 6 downregulated miRNAs unique to the PDDM group (Figure 1B). To assess confounding effects, we performed correlation analysis between DE-miRs and clinical indicators including HbA1c and CRP (Figure 1C). Several miRNAs showed strong correlations with glycemic and inflammatory markers but negligible association with age or BMI. This suggests that their expression changes are primarily linked to hyperglycemia and systemic inflammation, key features of diabetes.

**Table 2.** List of differentially expressed microRNAs (DEmiRs).

	miRNA	logFC	AveExpr	t	P.Value	adj.P.Val	B
PD upregulated	hsa-miR-4772-3p	−2.44578	1.500069	−3.95018	0.000474	0.908158	−3.07077
	hsa-miR-874-3p	−2.59805	4.348474	−3.54965	0.001372	0.912914	−4.10871
	hsa-miR-29c-5p	−2.42953	4.012643	−3.54873	0.001375	0.912914	−4.11106
	hsa-miR-532-3p	−2.25395	5.875374	−2.87719	0.007554	1	−5.74437
	hsa-miR-148b-3p	−2.20382	4.989199	−2.66832	0.012481	1	−6.21567
	hsa-miR-26a-2-3p	−2.25276	5.775102	−2.32803	0.027283	1	−6.93619
	hsa-miR-363-3p	−2.2995	5.817392	−2.30536	0.028691	1	−6.98189
PDDM upregulated	hsa-miR-30e-5p	−5.31111	5.344581	−6.12156	1.48E-06	0.003599	1.932526
	hsa-miR-451a	−2.88028	13.52685	−5.37338	1.09E-05	0.007214	−0.08149
	hsa-miR-144-5p	−2.68149	10.41072	−5.09008	2.33E-05	0.009017	−0.84804
	hsa-miR-16-5p	−2.18861	12.62435	−5.08288	2.38E-05	0.009017	−0.8675
	hsa-miR-363-3p	−4.1384	6.599435	−4.61573	8.39E-05	0.020247	−2.12769
	hsa-miR-20b-5p	−2.30937	8.510666	−4.44563	0.000133	0.021822	−2.58337
	hsa-miR-144-3p	−2.75415	11.15304	−4.42769	0.000139	0.021822	−2.63129
	hsa-miR-15a-5p	−3.63392	5.968102	−4.41609	0.000144	0.021822	−2.66226
	hsa-miR-140-3p	−2.1837	9.209531	−4.40816	0.000147	0.021822	−2.6834
	hsa-miR-4732-5p	−4.01011	5.759574	−4.405	0.000148	0.021822	−2.69182
PDDM downregulated	hsa-miR-432-5p	4.781511	5.274716	4.234996	0.000233	0.030193	−3.14372
	hsa-miR-32-3p	4.12088	6.118896	4.226469	0.000239	0.030193	−3.16629
	hsa-miR-556-3p	3.652303	8.710727	4.156234	0.000288	0.033261	−3.35186
	hsa-miR-664a-5p	4.346103	4.36033	4.132554	0.000307	0.033955	−3.41426
	hsa-miR-766-5p	3.434076	2.961078	4.037428	0.000395	0.039545	−3.66412
	hsa-miR-132-3p	2.977957	2.555604	4.031099	0.000402	0.039545	−3.68069

To focus on PDDM-specific miRNAs, common miRNAs shared between PDDM and PD (hsa-miR-363-3p) were excluded, resulting in nine upregulated and six downregulated PDDM miRNAs.

In addition, multiple regression analysis was performed across the three groups (control, PD, and PDDM) with adjustment for age and BMI (Supplementary Table 1). The results were largely consistent with the DEG analysis. In particular, regression analysis comparing controls and PDDM demonstrated robust upregulation of hsa-miR-30e-5p, hsa-miR-451a, hsa-miR-144-5p, and hsa-miR-16-5p, while most downregulated miRNAs—except for hsa-miR-556-3p and hsa-miR-132-3p—showed consistent decreases, thereby strengthening their potential as PDDM-specific markers. Furthermore, regression analysis comparing controls and PD revealed no significant alterations in miRNA expression, suggesting that dysregulation predominantly arises when periodontitis coexists with diabetes (PDDM). Collectively, these findings indicate that circulating miRNA expression changes are primarily attributable to group differences, with the most pronounced effects observed in PDDM, whereas the influence of age and BMI appears minimal.

Target genes of the DE-miRs were predicted using TargetScan, resulting in 216 upregulated and 64 downregulated target genes (Figure 1D; Supplementary Tables 2 and 3). GO network analysis revealed significant associations with diabetes-related processes such as glucose import, insulin receptor binding, and regulation of glycolytic process (Figure 1E). Notably, the genes enriched in Figure 1E are targets of the upregulated miRNAs and are therefore likely downregulated at the mRNA level. This inverse relationship suggests that these miRNAs may suppress critical components of glucose metabolism and insulin signaling, contributing to systemic metabolic dysregulation observed in diabetes. These results highlight the regulatory role of circulating miRNAs in disrupting key homeostatic pathways in PDDM.

**Table 3.** Common target genes identified from the miRNAs were consistently upregulated (22 genes) or downregulated (6 genes) across the GSE datasets. These genes were identified in at least four GSE datasets. These data were based on the official HGNC symbols, ensembl gene IDs, gene descriptions, and HGNC accession numbers.

	Symbol	Ensembl ID	Description	HGNC_Acc
up	*ARMC8*	ENSG00000114098	armadillo repeat containing 8	24999
	*CAAP1*	ENSG00000120159	caspase activity and apoptosis inhibitor 1	25834
	*CDC37L1*	ENSG00000106993	cell division cycle 37 like 1, HSP90 cochaperone	17179
	*CHD9*	ENSG00000177200	chromodomain helicase DNA binding protein 9	25701
	*CPEB3*	ENSG00000107864	cytoplasmic polyadenylation element binding protein 3	21746
	*CREBRF*	ENSG00000164463	CREB3 regulatory factor	24050
	*DAZAP2*	ENSG00000183283	DAZ associated protein 2	2684
	*ETNK1*	ENSG00000139163	ethanolamine kinase 1	24649
	*GOLGA4*	ENSG00000144674	golgin A4	4427
	*KPNA3*	ENSG00000102753	karyopherin subunit alpha 3	6396
	*PAFAH1B1*	ENSG00000007168	platelet activating factor acetylhydrolase 1b regulatory subunit 1	8574
	*RASGEF1B*	ENSG00000138670	RasGEF domain family member 1B	24881
	*SEMA6D*	ENSG00000137872	semaphorin 6D	16770
	*SETD3*	ENSG00000183576	SET domain containing 3, actin N3(tau)-histidine methyltransferase	20493
	*SLC25A36*	ENSG00000114120	solute carrier family 25 member 36	25554
	*SLC39A10*	ENSG00000196950	solute carrier family 39 member 10	20861
	*SNX16*	ENSG00000104497	sorting nexin 16	14980
	*SREK1*	ENSG00000153914	splicing regulatory glutamic acid and lysine rich protein 1	17882
	*SRP72*	ENSG00000174780	signal recognition particle 72	11303
	*TBL1XR1*	ENSG00000177565	TBL1X/Y related 1	29529
	*TRIP11*	ENSG00000100815	thyroid hormone receptor interactor 11	12305
	*ZBTB20*	ENSG00000181722	zinc finger and BTB domain containing 20	13503
down	*GABRA4*	ENSG00000109158	gamma-aminobutyric acid type A receptor subunit alpha4	4078
	*GLIPR1*	ENSG00000139278	GLI pathogenesis related 1	17001
	*KCNN3*	ENSG00000143603	potassium calcium-activated channel subfamily N member 3	6292
	*PAX5*	ENSG00000196092	paired box 5	8619
	*SLC24A2*	ENSG00000155886	solute carrier family 24 member 2	10976
	*SORT1*	ENSG00000134243	sortilin 1	11186

### 3.4. Validation using bulk RNA sequencing datasets from DM foot

**Figure 2.**
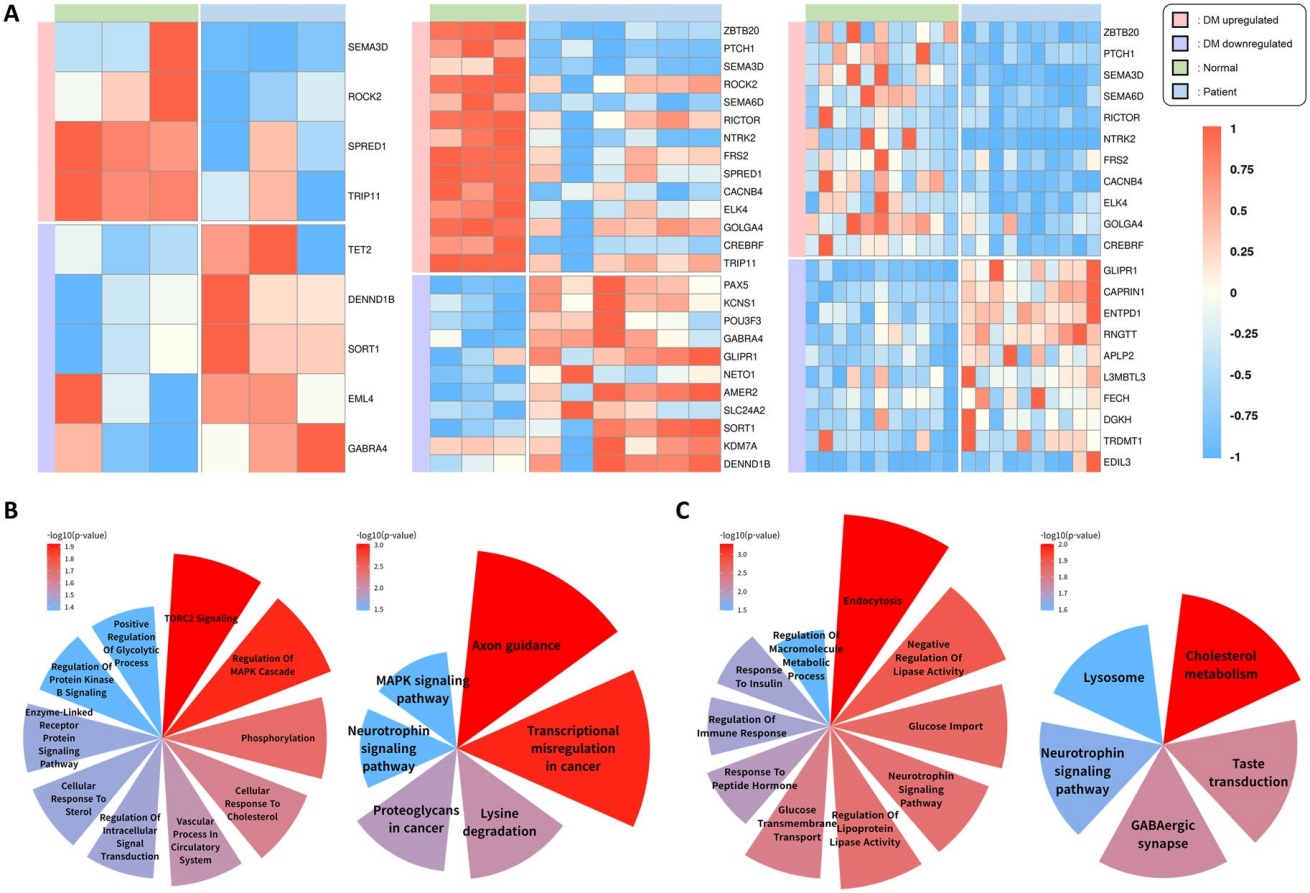
Differential expression and functional enrichment analysis of miRNA target genes in diabetic foot. (A) Heatmap of DEGs among predicted target genes of common miRNAs across three datasets (GSE68183, GSE80178, GSE199939). Genes included in the heatmap exhibited consistent differential expression between diabetic and control samples (student’s t-test, threshold *p* < 0.1; significance levels: *: *p* < 0.05; **: *p* < 0.01; ***: *p* < 0.001) and were annotated with enriched GO:BP or KEGG terms related to diabetes. (B, C) Polar area charts of GO:BP and KEGG enrichment analyses for upregulated (B) and downregulated (C) miRNA target genes. In both charts, GO:BP terms are shown on the left and KEGG pathways on the right. Only enrichment terms with p-values below 0.05 were included in the visualization. The red-to-blue color gradient reflects enrichment significance, expressed as –log₁。[*p*]).

Diabetic foot ulcers are severe complications of diabetes, primarily driven by chronic hyperglycemia, impaired circulation, neuropathy, and delayed wound healing [21,22]. To validate the biological relevance of the miRNA–mRNA regulatory network, we identified DEGs among predicted miRNA target genes across three bulk RNA-seq datasets derived from diabetic foot tissue (Figure 2A; Supplementary Table 4). To validate our findings, DEGs were identified from the predicted miRNA target genes using three foot-related GSE datasets (Figure 2A; Supplementary Table 4). Genes consistently altered in at least two datasets were subjected to Gene Ontology Biological Process (GOBP) and KEGG pathway enrichment analyses. The enriched terms were visualized using polar area charts, highlighting biological processes associated with diabetic foot pathology (Figure 2B and Figure 2C; Supplementary Table 5).

Target genes of upregulated miRNAs, which showed higher expression in non-diabetic samples, were significantly enriched in pathways related to glucose metabolism, lipid homeostasis, vascular function, and neurotrophic signaling. These results suggest that suppression of these genes may contribute to tissue dysfunction associated with diabetic complications. Among these, *ZBTB20*, a regulator of hepatic glucose homeostasis, was enriched in positive regulation of glycolytic process (GO:0045821) [23]. *PTCH1*, involved in of lipid homeostasis [24], was associated with cellular response to sterols and cholesterol (GO:0071397). It also participated in axon guidance (hsa04360), along with *SEMA3D*, *ROCK2*, and *SEMA6D*, which are essential for proper neural and vascular organization [25]. *ROCK2* was also enriched in vascular processes in the circulatory system (GO:0003018) and regulation of the MAPK cascade (GO:0043408), the latter in coordination with *NTRK2* and *SPRED1* [26]. *RICTOR*, involved in cellular metabolism and survival, supports insulin signaling and promotes tissue repair under diabetic stresss [27]. *RICTOR* is linked to the TORC2 signaling (GO:0038203) and collaborates with *NTRK2* in the regulation of protein kinase B signaling (GO:0051896). *NTRK2*, which also contributes to neuronal growth and survival [28], was enriched in the neurotrophin signaling pathway (hsa04722), functioning together with FRS2 to promote synaptic plasticity and neuroprotection. Additionally, *CACNB4*, a calcium channel regulator [29], was involved in MAPK signaling (hsa04010), likely *via* interactions with *ELK4* and *NTRK2*, contributing to cell survival and neuroprotection.

Conversely, target genes of downregulated miRNAs, which were upregulated in diabetic foot tissues, were enriched in pathways suggestive of compensatory responses to chronic inflammation and metabolic stress. For instance, *SORT1*, involved in lipid transport and LDL regulation, was enriched in glucose import (GO:0046323), response to insulin (GO:0032868), and cholesterol metabolism (hsa04979) [30]. *DENND1B*, associated with endocytosis (GO:0006897) and immune response regulation (GO:0050776), may modulate inflammation and promote tissue repair [31]. Additionally, *GABRA4*, a subunit of the GABA receptor family, contributes to calcium signaling and neuroprotection, and was enriched in GABAergic synapse (hsa04727), suggesting a role in neural adaptation under diabetic conditions [32].

### 3.5. Validation using bulk RNA sequencing datasets from DM nephropathy

**Figure 3.**
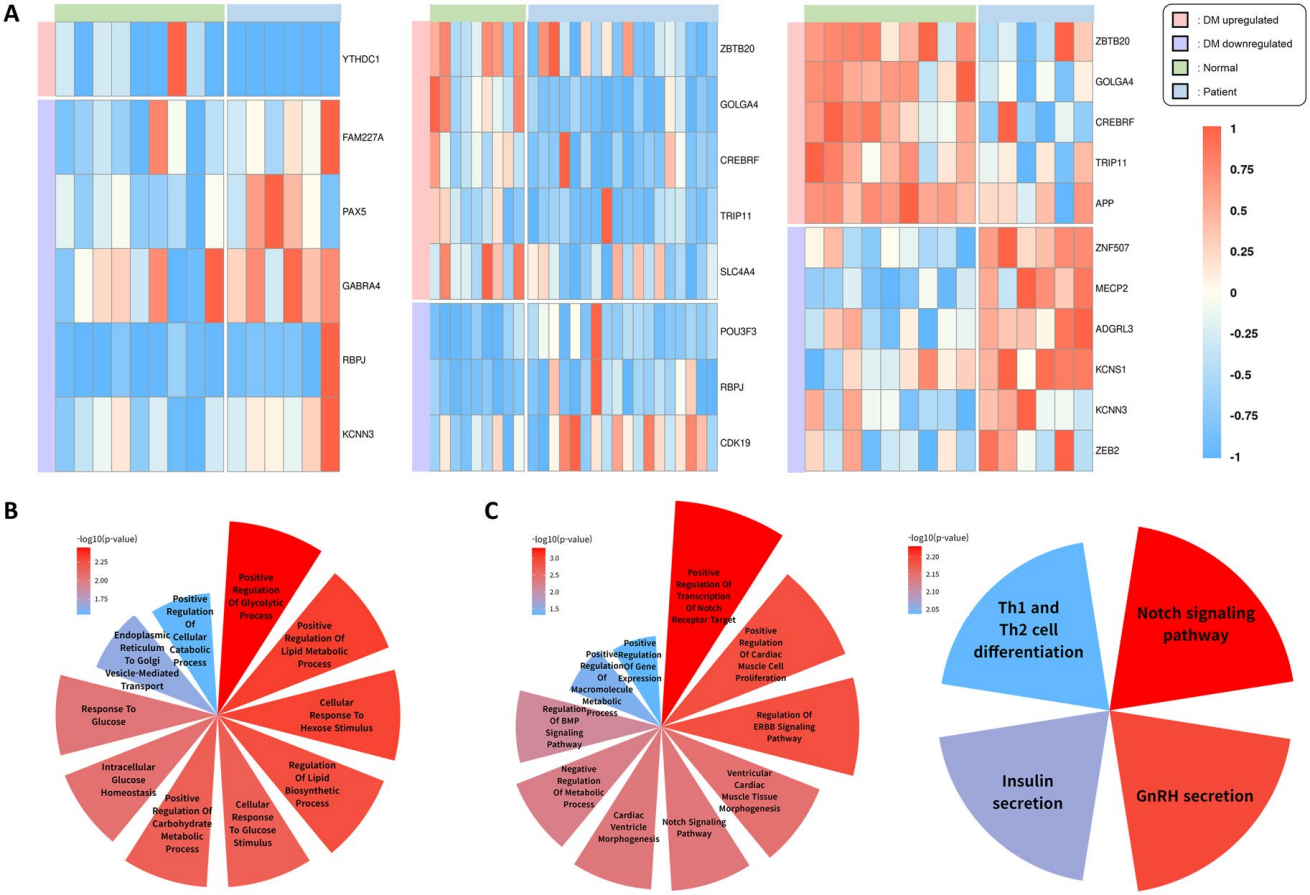
Differential expression and functional enrichment analysis of miRNA target genes in diabetic nephropathy. (A) Heatmap of DEGs among predicted target genes of common miRNAs across three datasets (GSE163603, GSE162830, GSE142025). Genes included in the heatmap exhibited consistent differential expression between diabetic and control samples (student’s t-test, threshold *p* < 0.1; significance levels: *: *p* < 0.05; **: *p* < 0.01; ***: *p* < 0.001) and were annotated with enriched GO:BP or KEGG terms related to diabetes. (B) Polar area chart of GO:BP enrichment analysis for upregulated miRNA target genes. Genes identified in at least two of the three datasets were included. Enriched GO:BP terms are related to renal function and diabetic complications. (C) Polar area chart of GO:BP and KEGG enrichment analyses for downregulated miRNA target genes. Genes identified in at least two datasets were included in the analysis. GO:BP terms are displayed on the left and KEGG pathways on the right. In both panels (B) and (C), only enrichment terms with p-values below 0.05 were included in the visualization. The red-to-blue color gradient reflects enrichment significance, expressed as –log₁。[*p*]).

Diabetic nephropathy is a prevalent and serious complication of diabetes, driven by chronic hyperglycemia, inflammation, and progressive renal damage [33]. To validate the relevance of miRNA–target gene interactions in the kidney, we analyzed DEGs predicted as miRNA targets using three bulk RNA-seq datasets related to diabetic kidney disease (Figure 3A; Supplementary Table 4). Genes commonly altered in at least two datasets were subjected to GOBP and KEGG pathway enrichment analyses, and the results were visualized using polar area charts (Figure 3B and Figure 3C; Supplementary Table 5).

Target genes of the upregulated miRNAs, including *GOLGA4, ZBTB20, CREBRF*, and *TRIP11*, were also identified in foot datasets, suggesting shared mechanisms across diabetic tissues. These genes were enriched in pathways related to glucose metabolism, inflammation regulation, and cellular stress response. For instance, *ZBTB20*, a metabolic transcription factor, was enriched in positive regulation of glycolytic process (GO:0045821) and lipid metabolic process (GO:0045834), contributing to glucose homeostasis and energy balance [34]. *GOLGA4* and *TRIP11*, involved in stabilizing Golgi structure and vesicular trafficking, were enriched in endoplasmic reticulum(ER)-to-Golgi vesicle-mediated transport (GO:0006888), promoting efficient protein processing and alleviating ER stress [35]. These findings indicate that suppression of such genes may impair metabolic adaptation and promote renal inflammation in diabetes.

Target genes of the downregulated miRNAs, such as *RBPJ*, a regulator of the Notch signaling pathway, facilitates cell proliferation and differentiation [36]. When overexpressed, it contributes to fibrosis and exacerbates kidney damage, related to processes regulating the ERBB (GO:1901184) and Notch signaling pathways (GO:0007219, hsa04330). Additionally, *KCNN3* regulates calcium signaling and vascular tone, contributing to vascular integrity while alleviating inflammation [37]. The association of *KCNN3* with the insulin (hsa04911) and GnRH secretion (hsa04929) indicates its role in maintaining endocrine and vascular health under diabetic conditions [38].

### 3.6. Validation using bulk RNA sequencing datasets DM pancreas

**Figure 4.**
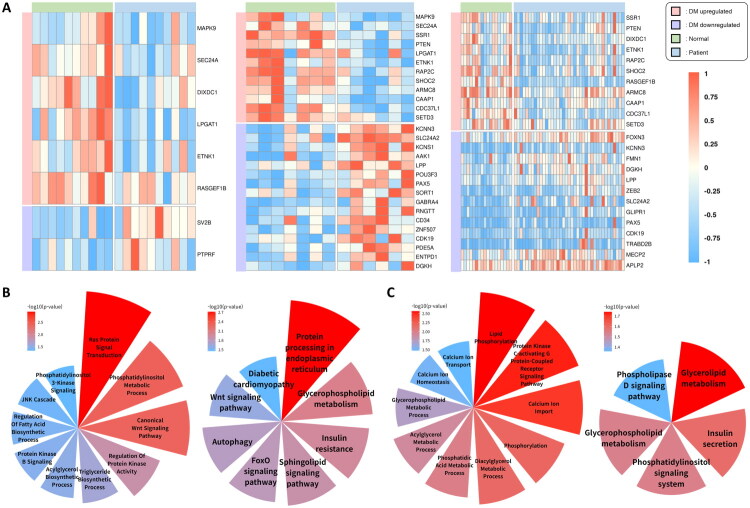
Differential expression and functional enrichment analysis of miRNA target genes in diabetic pancreas. (A) Heatmap of DEGs among predicted target genes of common miRNAs across three datasets (GSE20966, GSE25724, GSE164416). Genes included in the heatmap exhibited consistent differential expression between diabetic and control samples (student’s t-test, threshold *p* < 0.1; significance levels: *: *p* < 0.05; **: *p* < 0.01; ***: *p* < 0.001) and were annotated with enriched GO:BP or KEGG terms related to diabetes. (B, C) Polar area charts of GO:BP and KEGG enrichment analyses for upregulated (B) and downregulated (C) miRNA target genes: Genes identified in at least two of the three datasets were included in the enrichment analysis. In both charts, GO:BP terms are shown on the left and KEGG pathways on the right. Only enrichment terms with p-values below 0.05 were included in the visualization. The red-to-blue color gradient reflects enrichment significance, expressed as –log₁。[*p*]).

Pancreatic dysfunction in diabetes involves β-cell loss and impaired insulin secretion, contributing to chronic hyperglycemia. Given the central role of pancreatic tissue in glucose regulation, validating miRNA effects in this organ is essential for understanding systemic diabetic mechanisms. This process, exacerbated by inflammation and oxidative stress, disrupts glucose homeostasis and accelerates disease progression [39]. To investigate the regulatory impact of circulating miRNAs, we identified DEGs among predicted miRNA target genes using three pancreas-related bulk RNA-seq datasets (Figure 4A; Supplementary Table 4). Genes consistently altered in at least two datasets were subjected to GOBP and KEGG enrichment analyses, with results visualized using polar area charts (Figure 4B and Figure 4C; Supplementary Table 5).

Target genes of upregulated miRNAs—which were downregulated in diabetic pancreas tissues—were enriched in pathways related to protein processing, stress response, lipid metabolism, and cellular signaling. *MAPK9*, *SEC24A*, and *SSR1* contribute to protein translation, ER function, and vesicle transport, supporting β-cell insulin production. These genes were enriched in protein processing in the ER (hsa04141), with *MAPK9* additionally involved in the JNK cascade (GO:0007254), a major stress response pathway. Stress regulators *MAPK9* and *PTEN* were further enriched in insulin resistance (hsa04931), diabetic cardiomyopathy (hsa05415), and FoxO signaling (hsa04068). *PTEN* also modulated protein kinase B signaling (GO:0043491), phosphatidylinositol (PI) 3-kinase signaling (GO:0014065), and canonical Wnt signaling (GO:0060070), in coordination with *DIXDC1. LPGAT1* and *ETNK1*, enriched in glycerophospholipid metabolism (hsa00564), maintain lipid homeostasis by stabilizing phospholipid composition [40,41]. *LPGAT1* was also involved in PI metabolism (GO:0046488) and fatty acid biosynthesis regulation (GO:0042304), potentially cooperating with *PTEN* in lipid signaling modulation. *RAP2C*, *SHOC2*, and *RASGEF1B* are involved in cellular communication and repair mechanisms and were enriched in Ras protein signal transduction (GO:0007265). Additionally, genes such as *ARMC8*, *CAAP1*, *CDC37L1*, and *SETD3*, commonly upregulated in both pancreas and foot datasets, were linked to cytoskeletal stabilization, protein folding, and apoptosis regulation—suggesting shared systemic protective mechanisms across diabetic tissues.

In contrast, target genes of downregulated miRNAs, which were upregulated in diabetic samples, likely represent compensatory responses to hyperglycemia, calcium imbalance, and lipid dysregulation. *KCNN3* and *SLC24A2*, key calcium regulators, were enriched in insulin s secretion (hsa04911) and enhance calcium-mediated insulin secretion in β-cells [38,42]. *LPP*, a cytoskeletal and adhesion molecule, supports tissue remodeling under diabetic conditions [43]. *PAX5* and *CDK19* promote cell proliferation and differentiation, potentially facilitating pancreatic regeneration under metabolic stress [44,45]. *DGKH*, enriched in diacylglycerol(DAG) metabolism (GO:0046339), glycerolipid metabolism (GO:0006650), and PI signaling (hsa04070), regulates lipid balance and mitigates DAG-induced insulin resistance [46]. Its involvement in these pathways underscores a compensatory mechanism aimed at restoring lipid signaling homeostasis in diabetes.

### 3.7. Validation using bulk RNA sequencing datasets from DM retinopathy

**Figure 5.**
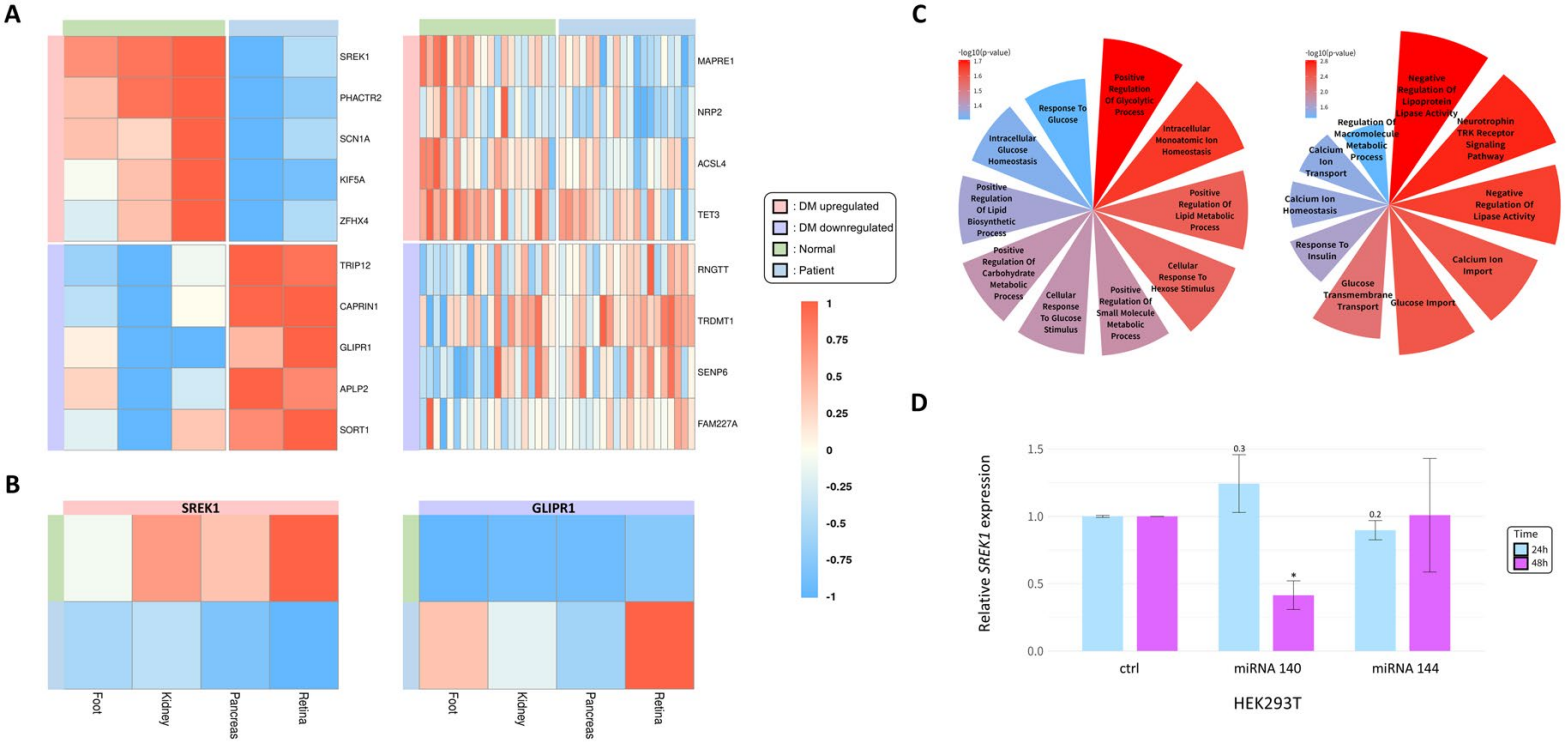
Diabetic retinopathy-specific gene expression and cross-organ validation of common miRNA targets. (A) Heatmap of DEGs among predicted target genes of common miRNAs across two datasets (GSE102485, GSE160306). However, due to the limited number of available datasets for diabetic retinal tissue, DEG patterns were inconsistent between the two datasets, and GO:BP and KEGG enrichment analyses could not be performed. (B) Cross-organ miRNA target genes: *SREK1* (upregulated) and *GLIPR1* (downregulated) were consistently identified as miRNA targets across all four organs—foot, kidney, pancreas, and retina. (C) GO:BP enrichment analysis of frequently identified genes: GO:BP terms enriched among upregulated (left) and downregulated (right) target genes that appeared in four or more of the 11 datasets are visualized. Only enrichment terms with p-values below 0.05 were included in the visualization. The red-to-blue color gradient reflects enrichment significance, expressed as –log₁。[*p*]). (D) *SREK1* mRNA expression in HEK293T cells following miRNA mimic transfection. A significant reduction was observed with miR-140 at 48 h (student’s t-test vs. negative control mimic; significance levels: *p < 0.05; **p < 0.01; ***p < 0.001).

Diabetic retinopathy is a severe complication of diabetes characterized by progressive damage to the retinal vasculature and neuronal tissues, ultimately resulting in vision impairment and blindness in advanced stages. This pathological process, driven by chronic hyperglycemia, inflammation, and oxidative stress, disrupts retinal homeostasis and accelerates disease progression [47]. To explore the regulatory impact of miRNAs in diabetic retinal dysfunction, DEGs were identified based on predicted miRNA target genes using two independent retina-related bulk RNA-seq datasets (Figure 5A; Supplementary Table 4). However, DEG patterns were not consistent between the two datasets, which may reflect the limited number and heterogeneity of available transcriptomic data for diabetic retinal tissue.

### 3.8. Common target genes and GOBP enrichment across four organs

A cross-organ analysis identified consistently regulated target genes across datasets. Among them, *SREK1* was identified as a target of upregulated miRNAs, and *GLIPR1* as a target of downregulated miRNAs across foot, kidney, pancreas, and retina datasets (Figure 5B).

*SREK1* encodes a nuclear RNA-binding protein involved in RNA splicing and mRNA processing. According to NCBI Gene, it regulates alternative splicing by modulating the activity of other splicing factors. *SREK1* is predominantly localized to nuclear speckles and is widely expressed across human tissues, supporting its role in post-transcriptional gene regulation. Recent studies have linked *SREK1* dysfunction to metabolic disorders, including obesity, type 2 diabetes, and hepatic steatosis [48,49].

In contrast, *GLIPR1* encodes a p53-inducible immune-modulatory protein involved in the regulation of inflammation, oxidative stress responses, and apoptotic signaling. It has been shown to promote reactive oxygen species (ROS)-mediated cell death and suppress oncogenic pathways such as c-Myc [50]. In addition, *GLIPR1* has been implicated in the regulation of epithelial–mesenchymal transition (EMT), cancer cell motility, and anti-tumor immune responses through pathways such as PI3K/PDK1/ROCK1 [51].

Additionally, we identified genes that appeared in four or more datasets among the targets of upregulated and downregulated miRNAs, resulting in 22 and 6 genes, respectively (Table 3). GOBP enrichment analysis was performed to characterize these genes (Figure 5C). All genes listed in Table 3 were consistently detected in foot, kidney, and pancreas datasets, underscoring their potential relevance to diabetes pathophysiology. Their recurrence across multiple organs and datasets strengthens their reliability as candidate markers or regulators of diabetic complications.

### 3.9. Experimental validation of *SREK1* regulation by miR-140 and miR-144 in HEK293T cells

To validate the regulatory relationship between upregulated circulating miRNAs and their predicted target gene *SREK1*, we performed transfection experiments in HEK293T cells. Cells were treated with synthetic mimics of hsa-miR-140-3p and hsa-miR-144-3p, both previously identified as upregulated in the PDDM group. Among these, miR-140 was selected as the representative mimic for functional validation.

Total RNA was extracted at 24 and 48 h after transfection, followed by cDNA synthesis and quantitative real-time PCR analysis. The sequences of the miRNA mimics and the primers used for quantifying *SREK1* mRNA and mature miRNAs are listed in Supplementary Tables 6–8, respectively.

*SREK1* mRNA expression was significantly reduced in cells treated with the miR-140 mimic, especially at 48 h post-transfection (Figure 5D). In contrast, cells treated with the miR-144 mimic showed no consistent reduction in *SREK1* expression. These results demonstrate that miR-140 more effectively regulates *SREK1* post-transcriptionally in HEK293T cells, supporting its functional role in target gene regulation *in vitro*.

## 4. Discussion

This study investigated the systemic regulatory role of circulating exosomal miRNAs in diabetes, focusing on both organ-specific and shared functions across multiple organs. Unlike previous studies that analyzed miRNAs within a single tissue or without excluding comorbid inflammation, our approach integrates plasma miRNA profiles with bulk RNA-seq data from the foot, kidney, pancreas, and retina. This enabled identification of miRNA–mRNA networks that contribute to organ-specific and systemic mechanisms of diabetic complications.

Functionally, the target genes of upregulated miRNAs were generally more highly expressed in healthy controls and were significantly enriched in pathways related to glucose metabolism, lipid homeostasis, stress responses, and tissue repair. These genes appear to maintain physiological functions under non-diabetic conditions, and their suppression may reduce cellular resilience under diabetic stress. In contrast, target genes of downregulated miRNAs exhibited increased expression in diabetic tissues and were enriched in inflammatory, oxidative, and compensatory signaling pathways, potentially reflecting adaptive responses to chronic hyperglycemia and metabolic imbalance.

Organ-specific analyses provided further insight. In the foot, upregulated targets were involved in glycolysis, vascular integrity, and axon guidance, supporting wound healing and neurovascular maintenance. Downregulated targets were linked to immune regulation and lipid transport, possibly reflecting local reparative processes. In the kidney, upregulated targets were involved in vesicle trafficking and ER stress response, while downregulated targets contributed to calcium signaling and vascular tone—key to renal function. In the pancreas, upregulated targets promoted insulin secretion, protein processing, and phospholipid metabolism, contributing to β-cell survival. Downregulated targets included regulators of DAG and PI signaling, mitigating insulin resistance and lipotoxic stress. In the case of the retina, consistent DEGs were not observed between datasets, possibly reflecting current limitations in dataset size and heterogeneity. With the accumulation of high-quality transcriptomic data in diabetic retina, future analyses may allow more robust validation and improved mechanistic understanding of retinal dysfunction.

Despite these organ-level differences, common regulatory signatures emerged. Upregulated miRNA targets were consistently associated with protein synthesis, cytoskeletal stability, and metabolic adaptation, indicating broad protective functions. Downregulated targets were enriched in inflammation, tissue remodeling, and stress response pathways, consistent with diabetes-related pathophysiology.

A cross-organ analysis further identified *SREK1* and *GLIPR1* as common target genes across all four tissues. *SREK1* is a splicing regulator that modulates alternative pre-mRNA processing and plays a pivotal role in post-transcriptional gene regulation [49]. Emerging evidence indicates that *SREK1* contributes to the expression of small nucleolar RNAs, including *SNORD115* and *SNORD116*, which are closely associated with neuroendocrine function and metabolic control [48]. Although these findings have primarily been linked to neurodevelopmental phenotypes, concurrent metabolic abnormalities—such as insulin resistance and impaired energy homeostasis—suggest that *SREK1* may also influence hypothalamic and endocrine pathways relevant to diabetes. In addition, isoform switching of *SREK1* has been implicated in hepatocellular carcinoma progression through the modulation of nonsense-mediated decay and competing endogenous RNA networks. Collectively, these findings support the notion that *SREK1* serves as a molecular link between transcriptomic dysregulation and metabolic disease, including diabetes-related complications. To experimentally verify the regulatory influence of miRNAs on *SREK1* expression, we performed miRNA mimic transfection in HEK293T cells. The results demonstrated that miR-140 significantly reduced *SREK1* mRNA expression, particularly at 48 h post-transfection, whereas miR-144 did not exert a consistent effect. These findings experimentally support the regulatory relationship between miR-140 and *SREK1*, suggesting that miR-140 may function as a more effective and specific post-transcriptional modulator of *SREK1* in this cellular model.

Another key gene, *GLIPR1,* encodes a p53-inducible immune-modulatory protein that plays diverse roles in inflammation, oxidative stress responses, and apoptosis [52]. In cancer contexts, *GLIPR1* has been shown to promote tumor proliferation, metastasis, and chemoresistance. Notably, in hepatocellular carcinoma, *GLIPR1* activates the PI3K/PDK1/ROCK1 signaling axis, thereby enhancing EMT, cellular motility, and resistance to 5-fluorouracil [51]. In prostate cancer, systemic administration of a truncated recombinant form (*GLIPR1*-ΔTM) has demonstrated potent anti-tumor effects, including induction of ROS-mediated apoptosis and suppression of oncogenic c-Myc signaling. Beyond oncology, *GLIPR1* has also been implicated in metabolic regulation, as genetic variation in animal models has been associated with fat deposition and energy balance. These findings suggest that *GLIPR1* functions as a context-dependent regulator of cell fate, with roles in tumor biology, immune modulation, and metabolic homeostasis [50]. Its multifaceted actions may also be relevant to chronic diseases characterized by inflammation and metabolic imbalance, such as diabetes and its complications. The consistent appearance of both genes across multiple organs supports their potential as systemic biomarkers or therapeutic targets.

Importantly, our findings reinforce the endocrine-like role of circulating exosomal miRNAs in regulating gene expression across distant tissues. The miRNA–mRNA regulatory pairs identified in this study include both computationally predicted interactions—derived from TargetScan and further interpreted through GO and KEGG enrichment analyses—and experimentally supported interactions, such as the regulatory relationship between miR-144, miR-140 and *SREK1*. Nonetheless, several of these associations remain putative and require further experimental validation to confirm their biological significance. Expansion of mechanistic studies employing diverse miRNA mimics or inhibitors may further elucidate the causal roles of key miRNAs in regulating target genes such as *SREK1* and *GLIPR1*. Such approaches could determine whether targeted modulation of miRNA expression can reverse dysregulated gene regulation and alleviate tissue injury associated with diabetes-related complications. Collectively, these insights provide a strong foundation for miRNA-based therapeutic strategies aimed at resolving tissue-specific metabolic and inflammatory dysregulation. The identification of PDDM-specific miRNAs and their regulatory networks may offer new directions for the diagnosis and treatment of diabetes and its multi-organ complications.

A key contextual consideration in our study is the absence of a diabetes-only control group, as all diabetic patients available for sampling also presented with periodontitis. Rather than excluding this commonly co-occurring condition, we designed the study to capture miRNA regulatory patterns characteristic of patients with both diabetes and periodontitis (PDDM), reflecting clinically relevant conditions. Follow-up study is planned to include diabetes-only patients, allowing us to better distinguish the individual and combined effects of these two diseases. Additionally, examining how the identified miRNAs contribute specifically to periodontitis, independent of their role in diabetes, will be a valuable extension of this work.

In this study, DE-miRNAs were identified using an unadjusted p-value threshold of 0.05, and a cutoff of *p* < 0.1 was applied for DEG validation in tissue-level analyses. This exploratory approach was intended to capture a broader range of biologically meaningful biomarker candidates, considering the complexity of systemic regulation in diabetes and the limitations in sample size. Despite the relatively relaxed criteria, we sought to minimize the risk of false positives and enhance the robustness of our findings by validating the expression patterns of predicted target genes across multiple independent RNA-Seq cohorts. Only miRNA–mRNA pairs that were consistently observed across multiple tissues were included in the final analysis. Although the lack of multiple testing correction may be viewed as a limitation, the primary aim of this study was to explore biologically relevant regulatory signals during the discovery phase and to establish a foundation for future investigation. In subsequent research, we plan to refine and validate the pathological relevance of the identified candidates through quantitative analysis in larger and more homogeneous cohorts, as well as expanded cross-tissue transcriptomic comparisons.

Given the inherent fragmentation of exosomal RNA, RNA integrity could not be formally assessed; however, RNA quality and size distribution were evaluated using standard protocols. Additional biophysical or molecular characterization of exosomes, including particle sizing and surface marker profiling, was not performed. Accordingly, the term “exosomal RNA” is used based on the isolation protocol, in accordance with the MISEV2018 guidelines and commonly accepted practices in extracellular RNA research. In addition, exosomal treatment experiments were not performed. Establishing reliable in-vitro conditions that accurately mimic exosome–cell interactions remains technically challenging. Future studies will be required to determine whether exosome-delivered miRNAs, such as miR-144 and miR-140, can modulate the proposed targets in a physiologically relevant context. These considerations, along with plans for validation in larger, more homogeneous cohorts, will contribute to the elucidation and validation of exosomal miRNA signatures as systemic biomarkers for diabetes-related complications.

## 5. Conclusion

This study highlights the systemic role of circulating exosomal miRNAs in diabetes by identifying PDDM-specific miRNAs and their target genes across multiple organs. Upregulated and downregulated miRNAs showed distinct expression patterns linked to metabolic regulation, inflammation, and tissue repair. The identification of common targets such as *SREK1* and *GLIPR1* supports the potential of these miRNAs as systemic biomarkers and therapeutic targets. Our findings provide a foundation for miRNA-based strategies to better understand and manage diabetes and its complications.

## Supplementary Material

Supplemental Material.docx

## Data Availability

The sequence data generated in this study have been deposited in the NCBI Gene Expression Omnibus (GEO) under accession number GSE301956. For validation of systemic and organ-specific miRNA target genes, the following GEO datasets were utilized: GSE68183, GSE80178, and GSE199939 for foot; GSE142025, GSE162830, and GSE163603 for kidney; GSE20966, GSE25724, and GSE164416 for pancreas; and GSE102485 and GSE160306 for retina.
