# Supplementary material for: Identification of circulating miRNA alterations in diabetes patients excluding periodontitis effects: insights into target gene downregulation in diabetic complications
Source: Ann Med. 2025 Nov 10;57(1):2567609. doi: 10.1080/07853890.2025.2567609 (PMC12604137; doi:10.1080/07853890.2025.2567609)
Supplement: Supplemental Material.docx [file IANN_A_2567609_SM6426.docx]

**Supplementary Table 1.** Regression analysis results for PDDM-specific miRNAs. Multiple regression models were performed with adjustment for age and BMI as covariates. Analyses included three-group comparisons (control as the reference) as well as two-group contrasts (control vs. PD and control vs. PDDM). Statistical testing was conducted using linear regression, and p-values were adjusted using the false discovery rate (FDR) method.

| **3-group** | **miRNA** | **group** | **estimate** | **std.error** | **statistic** | **p.value** | **p.adj** |
| --- | --- | --- | --- | --- | --- | --- | --- |
| PDDM  upregulated | hsa-miR-30e-5p | PD | 41.2629 | 191.2063 | 0.2158 | 0.8311 | 0.9286 |
|  |  | PDDM | 736.9512 | 194.3334 | 3.7922 | 0.0010 | 0.0044 |
|  | hsa-miR-451a | PD | 667.2726 | 10130.1778 | 0.0659 | 0.9481 | 0.9728 |
|  |  | PDDM | 51039.1352 | 10295.8483 | 4.9573 | 0.0001 | 0.0006 |
|  | hsa-miR-144-5p | PD | -1328.8530 | 930.9656 | -1.4274 | 0.1675 | 0.8945 |
|  |  | PDDM | 3771.7888 | 946.1907 | 3.9863 | 0.0006 | 0.0042 |
|  | hsa-miR-16-5p | PD | 1941.2691 | 2888.3341 | 0.6721 | 0.5085 | 0.9286 |
|  |  | PDDM | 14771.9621 | 2935.5704 | 5.0321 | 0.0000 | 0.0006 |
|  | hsa-miR-20b-5p | PD | 701.5530 | 441.5676 | 1.5888 | 0.1264 | 0.8945 |
|  |  | PDDM | 1750.7378 | 448.7891 | 3.9010 | 0.0008 | 0.0042 |
|  | hsa-miR-144-3p | PD | -686.6676 | 3452.2473 | -0.1989 | 0.8442 | 0.9286 |
|  |  | PDDM | 10755.6289 | 3508.7059 | 3.0654 | 0.0057 | 0.0138 |
|  | hsa-miR-15a-5p | PD | -3.5838 | 103.9831 | -0.0345 | 0.9728 | 0.9728 |
|  |  | PDDM | 342.8555 | 105.6836 | 3.2442 | 0.0037 | 0.0108 |
|  | hsa-miR-140-3p | PD | 240.2180 | 551.4277 | 0.4356 | 0.6674 | 0.9286 |
|  |  | PDDM | 1534.0997 | 560.4458 | 2.7373 | 0.0120 | 0.0260 |
|  | hsa-miR-4732-5p | PD | 69.3900 | 114.7011 | 0.6050 | 0.5514 | 0.9286 |
|  |  | PDDM | 380.4353 | 116.5770 | 3.2634 | 0.0036 | 0.0108 |
| PDDM  downregulated | hsa-miR-432-5p | PD | 39.2458 | 130.0747 | 0.3017 | 0.7657 | 0.9286 |
|  |  | PDDM | -352.0871 | 132.2019 | -2.6633 | 0.0142 | 0.0260 |
|  | hsa-miR-32-3p | PD | -36.8976 | 124.0627 | -0.2974 | 0.7689 | 0.9286 |
|  |  | PDDM | -299.5083 | 126.0917 | -2.3753 | 0.0267 | 0.0451 |
|  | hsa-miR-556-3p | PD | 845.9111 | 967.1699 | 0.8746 | 0.3912 | 0.9286 |
|  |  | PDDM | -1367.3530 | 982.9872 | -1.3910 | 0.1781 | 0.2063 |
|  | hsa-miR-664a-5p | PD | 69.3213 | 64.9381 | 1.0675 | 0.2973 | 0.9286 |
|  |  | PDDM | -149.8820 | 66.0001 | -2.2709 | 0.0333 | 0.0523 |
|  | hsa-miR-766-5p | PD | -34.5518 | 24.0342 | -1.4376 | 0.1646 | 0.8945 |
|  |  | PDDM | -65.9551 | 24.4273 | -2.7001 | 0.0131 | 0.0260 |
|  | hsa-miR-132-3p | PD | 12.4618 | 32.6545 | 0.3816 | 0.7064 | 0.9286 |
|  |  | PDDM | -51.8506 | 33.1886 | -1.5623 | 0.1325 | 0.1619 |
| **2-group**  **(Control, PD)** | **miRNA** | **group** | **estimate** | **std.error** | **statistic** | **p.value** | **p.adj** |
| PDDM  upregulated | hsa-miR-30e-5p | PD | 82.5056 | 76.6163 | 1.0769 | 0.2997 | 0.6231 |
|  | hsa-miR-451a | PD | -1604.9602 | 7514.8414 | -0.2136 | 0.8340 | 0.9371 |
|  | hsa-miR-144-5p | PD | -1312.8518 | 687.4180 | -1.9098 | 0.0769 | 0.6018 |
|  | hsa-miR-16-5p | PD | 1017.0871 | 2817.7729 | 0.3610 | 0.7235 | 0.8843 |
|  | hsa-miR-20b-5p | PD | 677.0552 | 413.5286 | 1.6373 | 0.1238 | 0.6231 |
|  | hsa-miR-144-3p | PD | -149.2908 | 2144.5269 | -0.0696 | 0.9455 | 0.9833 |
|  | hsa-miR-15a-5p | PD | 36.5060 | 29.7274 | 1.2280 | 0.2397 | 0.6231 |
|  | hsa-miR-140-3p | PD | 476.3226 | 512.2523 | 0.9299 | 0.3682 | 0.6231 |
|  | hsa-miR-4732-5p | PD | 31.5508 | 30.4650 | 1.0356 | 0.3179 | 0.6231 |
| PDDM  downregulated | hsa-miR-432-5p | PD | 64.6129 | 149.7104 | 0.4316 | 0.6726 | 0.8834 |
|  | hsa-miR-32-3p | PD | -3.2845 | 154.0286 | -0.0213 | 0.9833 | 0.9833 |
|  | hsa-miR-556-3p | PD | 720.9224 | 1274.7420 | 0.5655 | 0.5807 | 0.8516 |
|  | hsa-miR-664a-5p | PD | 82.3096 | 82.0230 | 1.0035 | 0.3327 | 0.6231 |
|  | hsa-miR-766-5p | PD | -35.9852 | 31.9989 | -1.1246 | 0.2797 | 0.6231 |
|  | hsa-miR-132-3p | PD | 17.9902 | 43.0834 | 0.4176 | 0.6826 | 0.8834 |
| **2-group**  **(Control, PDDM)** | **miRNA** | **group** | **estimate** | **std.error** | **statistic** | **p.value** | **p.adj** |
| PDDM  upregulated | hsa-miR-30e-5p | PDDM | 683.9998 | 268.3870 | 2.5486 | 0.0255 | 0.0702 |
|  | hsa-miR-451a | PDDM | 46699.0242 | 12854.6050 | 3.6329 | 0.0034 | 0.0194 |
|  | hsa-miR-144-5p | PDDM | 3380.2105 | 1254.1622 | 2.6952 | 0.0195 | 0.0670 |
|  | hsa-miR-16-5p | PDDM | 13100.9892 | 3133.0212 | 4.1816 | 0.0013 | 0.0140 |
|  | hsa-miR-20b-5p | PDDM | 1643.9528 | 454.4898 | 3.6171 | 0.0035 | 0.0194 |
|  | hsa-miR-144-3p | PDDM | 9539.9071 | 4442.5215 | 2.1474 | 0.0529 | 0.0895 |
|  | hsa-miR-15a-5p | PDDM | 315.4032 | 143.7479 | 2.1941 | 0.0486 | 0.0895 |
|  | hsa-miR-140-3p | PDDM | 1321.7709 | 684.1951 | 1.9319 | 0.0773 | 0.1134 |
|  | hsa-miR-4732-5p | PDDM | 374.1770 | 162.9735 | 2.2959 | 0.0405 | 0.0895 |
| PDDM  downregulated | hsa-miR-432-5p | PDDM | -293.5044 | 142.2479 | -2.0633 | 0.0614 | 0.0965 |
|  | hsa-miR-32-3p | PDDM | -249.6040 | 114.8624 | -2.1731 | 0.0505 | 0.0895 |
|  | hsa-miR-556-3p | PDDM | -1496.5283 | 874.5903 | -1.7111 | 0.1128 | 0.1459 |
|  | hsa-miR-664a-5p | PDDM | -111.7422 | 64.6780 | -1.7277 | 0.1097 | 0.1459 |
|  | hsa-miR-766-5p | PDDM | -66.3941 | 30.3932 | -2.1845 | 0.0495 | 0.0895 |
|  | hsa-miR-132-3p | PDDM | -89.6996 | 12.6555 | -7.0878 | 0.0000 | 0.0003 |

**Supplementary Table 2.** Common target genes are upregulated in Periodontitis with Diabetes Mellitus (PDDM). These data were based on the official HGNC symbols, Ensembl gene IDs, gene descriptions, and HGNC accession numbers.

| **Symbol** | **Ensembl ID** | **Description** | **HGNC_Acc** |
| --- | --- | --- | --- |
| *ABHD2* | ENSG00000140526 | abhydrolase domain containing 2, acylglycerol lipase | 18717 |
| *ABL2* | ENSG00000143322 | ABL proto-oncogene 2, non-receptor tyrosine kinase | 77 |
| *ACSL4* | ENSG00000068366 | acyl-CoA synthetase long chain family member 4 | 3571 |
| *ACTR1A* | ENSG00000138107 | actin related protein 1A | 167 |
| *ACVR2B* | ENSG00000114739 | activin A receptor type 2B | 174 |
| *ADAM10* | ENSG00000137845 | ADAM metallopeptidase domain 10 | 188 |
| *ADAMTS3* | ENSG00000156140 | ADAM metallopeptidase with thrombospondin type 1 motif 3 | 219 |
| *ADAMTS5* | ENSG00000154736 | ADAM metallopeptidase with thrombospondin type 1 motif 5 | 221 |
| *ADAMTS6* | ENSG00000049192 | ADAM metallopeptidase with thrombospondin type 1 motif 6 | 222 |
| *AK4* | ENSG00000162433 | adenylate kinase 4 | 363 |
| *AMMECR1* | ENSG00000101935 | AMMECR nuclear protein 1 | 467 |
| *ANKIB1* | ENSG00000001629 | ankyrin repeat and IBR domain containing 1 | 22215 |
| *AP1S3* | ENSG00000152056 | adaptor related protein complex 1 subunit sigma 3 | 18971 |
| *APP* | ENSG00000142192 | amyloid beta precursor protein | 620 |
| *ARMC8* | ENSG00000114098 | armadillo repeat containing 8 | 24999 |
| *ATXN1L* | ENSG00000224470 | ataxin 1 like | 33279 |
| *AVL9* | ENSG00000105778 | AVL9 cell migration associated | 28994 |
| *BCL11B* | ENSG00000127152 | BCL11 transcription factor B | 13222 |
| *BRWD1* | ENSG00000185658 | bromodomain and WD repeat domain containing 1 | 12760 |
| *BTG2* | ENSG00000159388 | BTG anti-proliferation factor 2 | 1131 |
| *CAAP1* | ENSG00000120159 | caspase activity and apoptosis inhibitor 1 | 25834 |
| *CACNB4* | ENSG00000182389 | calcium voltage-gated channel auxiliary subunit beta 4 | 1404 |
| *CARNMT1* | ENSG00000156017 | carnosine N-methyltransferase 1 | 23435 |
| *CCNT2* | ENSG00000082258 | cyclin T2 | 1600 |
| *CDC37L1* | ENSG00000106993 | cell division cycle 37 like 1, HSP90 cochaperone | 17179 |
| *CDC42SE2* | ENSG00000158985 | CDC42 small effector 2 | 18547 |
| *CHD9* | ENSG00000177200 | chromodomain helicase DNA binding protein 9 | 25701 |
| *CHIC1* | ENSG00000204116 | cysteine rich hydrophobic domain 1 | 1934 |
| *CMTM4* | ENSG00000183723 | CKLF like MARVEL transmembrane domain containing 4 | 19175 |
| *CNOT6L* | ENSG00000138767 | CCR4-NOT transcription complex subunit 6 like | 18042 |
| *CPEB2* | ENSG00000137449 | cytoplasmic polyadenylation element binding protein 2 | 21745 |
| *CPEB3* | ENSG00000107864 | cytoplasmic polyadenylation element binding protein 3 | 21746 |
| *CPSF6* | ENSG00000111605 | cleavage and polyadenylation specific factor 6 | 13871 |
| *CREBRF* | ENSG00000164463 | CREB3 regulatory factor | 24050 |
| *CYB561D1* | ENSG00000174151 | cytochrome b561 family member D1 | 26804 |
| *DAZAP2* | ENSG00000183283 | DAZ associated protein 2 | 2684 |
| *DCUN1D1* | ENSG00000043093 | defective in cullin neddylation 1 domain containing 1 | 18184 |
| *DENND6A* | ENSG00000174839 | DENN domain containing 6A | 26635 |
| *DIXDC1* | ENSG00000150764 | DIX domain containing 1 | 23695 |
| *DLGAP1* | ENSG00000170579 | DLG associated protein 1 | 2905 |
| *DMTF1* | ENSG00000135164 | cyclin D binding myb like transcription factor 1 | 14603 |
| *DPP8* | ENSG00000074603 | dipeptidyl peptidase 8 | 16490 |
| *E2F3* | ENSG00000112242 | E2F transcription factor 3 | 3115 |
| *E2F7* | ENSG00000165891 | E2F transcription factor 7 | 23820 |
| *EIF5A2* | ENSG00000163577 | eukaryotic translation initiation factor 5A2 | 3301 |
| *ELAVL2* | ENSG00000107105 | ELAV like RNA binding protein 2 | 3313 |
| *ELK4* | ENSG00000158711 | ETS transcription factor ELK4 | 3326 |
| *ELL2* | ENSG00000118985 | elongation factor for RNA polymerase II 2 | 17064 |
| *EPHA7* | ENSG00000135333 | EPH receptor A7 | 3390 |
| *EPHB2* | ENSG00000133216 | EPH receptor B2 | 3393 |
| *ETNK1* | ENSG00000139163 | ethanolamine kinase 1 | 24649 |
| *EZH1* | ENSG00000108799 | enhancer of zeste 1 polycomb repressive complex 2 subunit | 3526 |
| *FAM199X* | ENSG00000123575 | family with sequence similarity 199, X-linked | 25195 |
| *FAM91A1* | ENSG00000176853 | family with sequence similarity 91 member A1 | 26306 |
| *FAT4* | ENSG00000196159 | FAT atypical cadherin 4 | 23109 |
| *FBXO33* | ENSG00000165355 | F-box protein 33 | 19833 |
| *FNDC3B* | ENSG00000075420 | fibronectin type III domain containing 3B | 24670 |
| *FRS2* | ENSG00000166225 | fibroblast growth factor receptor substrate 2 | 16971 |
| *GABRA1* | ENSG00000022355 | gamma-aminobutyric acid type A receptor subunit alpha1 | 4075 |
| *GLCE* | ENSG00000138604 | glucuronic acid epimerase | 17855 |
| *GOLGA1* | ENSG00000136935 | golgin A1 | 4424 |
| *GOLGA4* | ENSG00000144674 | golgin A4 | 4427 |
| *GRB10* | ENSG00000106070 | growth factor receptor bound protein 10 | 4564 |
| *GXYLT1* | ENSG00000151233 | glucoside xylosyltransferase 1 | 27482 |
| *HIPK2* | ENSG00000064393 | homeodomain interacting protein kinase 2 | 14402 |
| *HIPK3* | ENSG00000110422 | homeodomain interacting protein kinase 3 | 4915 |
| *HS3ST5* | ENSG00000249853 | heparan sulfate-glucosamine 3-sulfotransferase 5 | 19419 |
| *HTR2A* | ENSG00000102468 | 5-hydroxytryptamine receptor 2A | 5293 |
| *HTR4* | ENSG00000164270 | 5-hydroxytryptamine receptor 4 | 5299 |
| *IFFO2* | ENSG00000169991 | intermediate filament family orphan 2 | 27006 |
| *IGF2R* | ENSG00000197081 | insulin like growth factor 2 receptor | 5467 |
| *INO80D* | ENSG00000283510 | INO80 complex subunit D | 25997 |
| *INO80D* | ENSG00000114933 | INO80 complex subunit D | 25997 |
| *IRS1* | ENSG00000169047 | insulin receptor substrate 1 | 6125 |
| *ITPRIPL2* | ENSG00000205730 | ITPRIP like 2 | 27257 |
| *KIF5A* | ENSG00000155980 | kinesin family member 5A | 6323 |
| *KMT2A* | ENSG00000118058 | lysine methyltransferase 2A | 7132 |
| *KPNA1* | ENSG00000114030 | karyopherin subunit alpha 1 | 6394 |
| *KPNA3* | ENSG00000102753 | karyopherin subunit alpha 3 | 6396 |
| *LATS2* | ENSG00000150457 | large tumor suppressor kinase 2 | 6515 |
| *LCOR* | ENSG00000196233 | ligand dependent nuclear receptor corepressor | 29503 |
| *LGI2* | ENSG00000153012 | leucine rich repeat LGI family member 2 | 18710 |
| *LPGAT1* | ENSG00000123684 | lysophosphatidylglycerol acyltransferase 1 | 28985 |
| *LUZP1* | ENSG00000169641 | leucine zipper protein 1 | 14985 |
| *MAFK* | ENSG00000198517 | MAF bZIP transcription factor K | 6782 |
| *MAP3K13* | ENSG00000073803 | mitogen-activated protein kinase kinase kinase 13 | 6852 |
| *MAP3K9* | ENSG00000006432 | mitogen-activated protein kinase kinase kinase 9 | 6861 |
| *MAPK9* | ENSG00000050748 | mitogen-activated protein kinase 9 | 6886 |
| *MAPRE1* | ENSG00000101367 | microtubule associated protein RP/EB family member 1 | 6890 |
| *MBTPS2* | ENSG00000012174 | membrane bound transcription factor peptidase, site 2 | 15455 |
| *MDM4* | ENSG00000198625 | MDM4 regulator of p53 | 6974 |
| *MEX3C* | ENSG00000176624 | mex-3 RNA binding family member C | 28040 |
| *MMD* | ENSG00000108960 | monocyte to macrophage differentiation associated | 7153 |
| *MTMR3* | ENSG00000100330 | myotubularin related protein 3 | 7451 |
| *MYO5A* | ENSG00000197535 | myosin VA | 7602 |
| *MYO5B* | ENSG00000167306 | myosin VB | 7603 |
| *NACC2* | ENSG00000148411 | NACC family member 2 | 23846 |
| *NF1* | ENSG00000196712 | neurofibromin 1 | 7765 |
| *NRBP1* | ENSG00000115216 | nuclear receptor binding protein 1 | 7993 |
| *NRP2* | ENSG00000118257 | neuropilin 2 | 8005 |
| *NTRK2* | ENSG00000148053 | neurotrophic receptor tyrosine kinase 2 | 8032 |
| *NUFIP2* | ENSG00000108256 | nuclear FMR1 interacting protein 2 | 17634 |
| *ONECUT2* | ENSG00000119547 | one cut homeobox 2 | 8139 |
| *OTUD4* | ENSG00000164164 | OTU deubiquitinase 4 | 24949 |
| *PAFAH1B1* | ENSG00000007168 | platelet activating factor acetylhydrolase 1b regulatory subunit 1 | 8574 |
| *PAFAH1B2* | ENSG00000168092 | platelet activating factor acetylhydrolase 1b catalytic subunit 2 | 8575 |
| *PAG1* | ENSG00000076641 | phosphoprotein membrane anchor with glycosphingolipid microdomains 1 | 30043 |
| *PCDH17* | ENSG00000118946 | protocadherin 17 | 14267 |
| *PDE3B* | ENSG00000152270 | phosphodiesterase 3B | 8779 |
| *PDK3* | ENSG00000067992 | pyruvate dehydrogenase kinase 3 | 8811 |
| *PEAK1* | ENSG00000173517 | pseudopodium enriched atypical kinase 1 | 29431 |
| *PGM2L1* | ENSG00000165434 | phosphoglucomutase 2 like 1 | 20898 |
| *PHACTR2* | ENSG00000112419 | phosphatase and actin regulator 2 | 20956 |
| *PLAG1* | ENSG00000181690 | PLAG1 zinc finger | 9045 |
| *PLXNC1* | ENSG00000136040 | plexin C1 | 9106 |
| *POU2F1* | ENSG00000143190 | POU class 2 homeobox 1 | 9212 |
| *PPM1A* | ENSG00000100614 | protein phosphatase, Mg2+/Mn2+ dependent 1A | 9275 |
| *PPM1E* | ENSG00000175175 | protein phosphatase, Mg2+/Mn2+ dependent 1E | 19322 |
| *PSMD7* | ENSG00000103035 | proteasome 26S subunit, non-ATPase 7 | 9565 |
| *PTCH1* | ENSG00000185920 | patched 1 | 9585 |
| *PTEN* | ENSG00000284792 | phosphatase and tensin homolog | 9588 |
| *PTEN* | ENSG00000171862 | phosphatase and tensin homolog | 9588 |
| *PTPN4* | ENSG00000088179 | protein tyrosine phosphatase non-receptor type 4 | 9656 |
| *QKI* | ENSG00000112531 | QKI, KH domain containing RNA binding | 21100 |
| *RAB10* | ENSG00000084733 | RAB10, member RAS oncogene family | 9759 |
| *RAB11FIP1* | ENSG00000156675 | RAB11 family interacting protein 1 | 30265 |
| *RAB30* | ENSG00000137502 | RAB30, member RAS oncogene family | 9770 |
| *RAP2C* | ENSG00000123728 | RAP2C, member of RAS oncogene family | 21165 |
| *RAPH1* | ENSG00000173166 | Ras association (RalGDS/AF-6) and pleckstrin homology domains 1 | 14436 |
| *RARB* | ENSG00000077092 | retinoic acid receptor beta | 9865 |
| *RASGEF1B* | ENSG00000138670 | RasGEF domain family member 1B | 24881 |
| *REEP3* | ENSG00000165476 | receptor accessory protein 3 | 23711 |
| *RET* | ENSG00000165731 | ret proto-oncogene | 9967 |
| *RGMA* | ENSG00000182175 | repulsive guidance molecule BMP co-receptor a | 30308 |
| *RICTOR* | ENSG00000164327 | RPTOR independent companion of MTOR complex 2 | 28611 |
| *RLIM* | ENSG00000131263 | ring finger protein, LIM domain interacting | 13429 |
| *RNF111* | ENSG00000157450 | ring finger protein 111 | 17384 |
| *RNF213* | ENSG00000173821 | ring finger protein 213 | 14539 |
| *RNF217* | ENSG00000146373 | ring finger protein 217 | 21487 |
| *ROCK2* | ENSG00000134318 | Rho associated coiled-coil containing protein kinase 2 | 10252 |
| *RORA* | ENSG00000069667 | RAR related orphan receptor A | 10258 |
| *SALL1* | ENSG00000103449 | spalt like transcription factor 1 | 10524 |
| *SCN1A* | ENSG00000144285 | sodium voltage-gated channel alpha subunit 1 | 10585 |
| *SCN2A* | ENSG00000136531 | sodium voltage-gated channel alpha subunit 2 | 10588 |
| *SCN8A* | ENSG00000196876 | sodium voltage-gated channel alpha subunit 8 | 10596 |
| *SEC22C* | ENSG00000093183 | SEC22 homolog C, vesicle trafficking protein | 16828 |
| *SEC24A* | ENSG00000113615 | SEC24 homolog A, COPII coat complex component | 10703 |
| *SEMA3D* | ENSG00000153993 | semaphorin 3D | 10726 |
| *SEMA6D* | ENSG00000137872 | semaphorin 6D | 16770 |
| *SETD3* | ENSG00000183576 | SET domain containing 3, actin N3(tau)-histidine methyltransferase | 20493 |
| *SH3GLB1* | ENSG00000097033 | SH3 domain containing GRB2 like, endophilin B1 | 10833 |
| *SHOC2* | ENSG00000108061 | SHOC2 leucine rich repeat scaffold protein | 15454 |
| *SINHCAF* | ENSG00000276371 | SIN3-HDAC complex associated factor | 30702 |
| *SINHCAF* | ENSG00000139146 | SIN3-HDAC complex associated factor | 30702 |
| *SKIL* | ENSG00000136603 | SKI like proto-oncogene | 10897 |
| *SLC1A2* | ENSG00000110436 | solute carrier family 1 member 2 | 10940 |
| *SLC25A36* | ENSG00000114120 | solute carrier family 25 member 36 | 25554 |
| *SLC36A1* | ENSG00000123643 | solute carrier family 36 member 1 | 18761 |
| *SLC39A10* | ENSG00000196950 | solute carrier family 39 member 10 | 20861 |
| *SLC4A4* | ENSG00000080493 | solute carrier family 4 member 4 | 11030 |
| *SLC4A8* | ENSG00000050438 | solute carrier family 4 member 8 | 11034 |
| *SMAD1* | ENSG00000170365 | SMAD family member 1 | 6767 |
| *SMIM13* | ENSG00000224531 | small integral membrane protein 13 | 27356 |
| *SNTB2* | ENSG00000260873 | syntrophin beta 2 | 11169 |
| *SNTB2* | ENSG00000168807 | syntrophin beta 2 | 11169 |
| *SNX16* | ENSG00000104497 | sorting nexin 16 | 14980 |
| *SOBP* | ENSG00000112320 | sine oculis binding protein homolog | 29256 |
| *SOCS6* | ENSG00000170677 | suppressor of cytokine signaling 6 | 16833 |
| *SON* | ENSG00000159140 | SON DNA and RNA binding protein | 11183 |
| *SPRED1* | ENSG00000166068 | sprouty related EVH1 domain containing 1 | 20249 |
| *SREK1* | ENSG00000153914 | splicing regulatory glutamic acid and lysine rich protein 1 | 17882 |
| *SRP72* | ENSG00000174780 | signal recognition particle 72 | 11303 |
| *SSR1* | ENSG00000124783 | signal sequence receptor subunit 1 | 11323 |
| *STOX2* | ENSG00000173320 | storkhead box 2 | 25450 |
| *STXBP5* | ENSG00000164506 | syntaxin binding protein 5 | 19665 |
| *TAB3* | ENSG00000157625 | TGF-beta activated kinase 1 (MAP3K7) binding protein 3 | 30681 |
| *TAOK1* | ENSG00000160551 | TAO kinase 1 | 29259 |
| *TBL1XR1* | ENSG00000177565 | TBL1X/Y related 1 | 29529 |
| *TET3* | ENSG00000187605 | tet methylcytosine dioxygenase 3 | 28313 |
| *TFPI2* | ENSG00000105825 | tissue factor pathway inhibitor 2 | 11761 |
| *TGIF2* | ENSG00000118707 | TGFB induced factor homeobox 2 | 15764 |
| *TLE4* | ENSG00000106829 | TLE family member 4, transcriptional corepressor | 11840 |
| *TMCC1* | ENSG00000172765 | transmembrane and coiled-coil domain family 1 | 29116 |
| *TNRC6B* | ENSG00000100354 | trinucleotide repeat containing adaptor 6B | 29190 |
| *TP53INP1* | ENSG00000164938 | tumor protein p53 inducible nuclear protein 1 | 18022 |
| *TRDN* | ENSG00000186439 | triadin | 12261 |
| *TRIP11* | ENSG00000100815 | thyroid hormone receptor interactor 11 | 12305 |
| *UBE2J1* | ENSG00000198833 | ubiquitin conjugating enzyme E2 J1 | 17598 |
| *UBE2V1* | ENSG00000244687 | ubiquitin conjugating enzyme E2 V1 | 12494 |
| *UBE3C* | ENSG00000009335 | ubiquitin protein ligase E3C | 16803 |
| *UBN2* | ENSG00000157741 | ubinuclein 2 | 21931 |
| *UNC5D* | ENSG00000156687 | unc-5 netrin receptor D | 18634 |
| *UNC80* | ENSG00000144406 | unc-80 homolog, NALCN channel complex subunit | 26582 |
| *USP31* | ENSG00000103404 | ubiquitin specific peptidase 31 | 20060 |
| *USP38* | ENSG00000170185 | ubiquitin specific peptidase 38 | 20067 |
| *WASL* | ENSG00000106299 | WASP like actin nucleation promoting factor | 12735 |
| *WEE1* | ENSG00000166483 | WEE1 G2 checkpoint kinase | 12761 |
| *WNK3* | ENSG00000196632 | WNK lysine deficient protein kinase 3 | 14543 |
| *XIRP2* | ENSG00000163092 | xin actin binding repeat containing 2 | 14303 |
| *XPR1* | ENSG00000143324 | xenotropic and polytropic retrovirus receptor 1 | 12827 |
| *YOD1* | ENSG00000180667 | YOD1 deubiquitinase | 25035 |
| *YTHDC1* | ENSG00000275272 | YTH N6-methyladenosine RNA binding protein C1 | 30626 |
| *YTHDC1* | ENSG00000083896 | YTH N6-methyladenosine RNA binding protein C1 | 30626 |
| *ZBTB18* | ENSG00000179456 | zinc finger and BTB domain containing 18 | 13030 |
| *ZBTB20* | ENSG00000181722 | zinc finger and BTB domain containing 20 | 13503 |
| *ZBTB34* | ENSG00000177125 | zinc finger and BTB domain containing 34 | 31446 |
| *ZBTB7A* | ENSG00000178951 | zinc finger and BTB domain containing 7A | 18078 |
| *ZC3H12C* | ENSG00000149289 | zinc finger CCCH-type containing 12C | 29362 |
| *ZCCHC2* | ENSG00000141664 | zinc finger CCHC-type containing 2 | 22916 |
| *ZDHHC21* | ENSG00000175893 | zinc finger DHHC-type palmitoyltransferase 21 | 20750 |
| *ZFHX4* | ENSG00000091656 | zinc finger homeobox 4 | 30939 |
| *ZMAT3* | ENSG00000172667 | zinc finger matrin-type 3 | 29983 |
| *ZNF264* | ENSG00000083844 | zinc finger protein 264 | 13057 |
| *ZNF275* | ENSG00000063587 | zinc finger protein 275 | 13069 |
| *ZNF362* | ENSG00000160094 | zinc finger protein 362 | 18079 |
| *ZNF367* | ENSG00000165244 | zinc finger protein 367 | 18320 |

**Supplementary Table 3.** Common target genes are downregulated in Periodontitis with Diabetes Mellitus (PDDM). These data were based on the official HGNC symbols, Ensembl gene IDs, gene descriptions, and HGNC accession numbers.

| **hgnc_symbol** | **ensembl_gene_id** | **description** | **HGNC_Acc** |
| --- | --- | --- | --- |
| *AAK1* | ENSG00000115977 | AP2 associated kinase 1 | 19679 |
| *ADGRL3* | ENSG00000150471 | adhesion G protein-coupled receptor L3 | 20974 |
| *AMER2* | ENSG00000165566 | APC membrane recruitment protein 2 | 26360 |
| *APC* | ENSG00000134982 | APC regulator of WNT signaling pathway | 583 |
| *APLP2* | ENSG00000084234 | amyloid beta precursor like protein 2 | 598 |
| *ARID1A* | ENSG00000117713 | AT-rich interaction domain 1A | 11110 |
| *BTAF1* | ENSG00000095564 | B-TFIID TATA-box binding protein associated factor 1 | 17307 |
| *CAPRIN1* | ENSG00000135387 | cell cycle associated protein 1 | 6743 |
| *CD34* | ENSG00000174059 | CD34 molecule | 1662 |
| *CDK19* | ENSG00000155111 | cyclin dependent kinase 19 | 19338 |
| *CNOT2* | ENSG00000111596 | CCR4-NOT transcription complex subunit 2 | 7878 |
| *DENND1B* | ENSG00000213047 | DENN domain containing 1B | 28404 |
| *DGKH* | ENSG00000102780 | diacylglycerol kinase eta | 2854 |
| *EDIL3* | ENSG00000164176 | EGF like repeats and discoidin domains 3 | 3173 |
| *EML4* | ENSG00000143924 | EMAP like 4 | 1316 |
| *ENTPD1* | ENSG00000138185 | ectonucleoside triphosphate diphosphohydrolase 1 | 3363 |
| *FAM222B* | ENSG00000173065 | family with sequence similarity 222 member B | 25563 |
| *FAM227A* | ENSG00000184949 | family with sequence similarity 227 member A | 44197 |
| *FECH* | ENSG00000066926 | ferrochelatase | 3647 |
| *FMN1* | ENSG00000248905 | formin 1 | 3768 |
| *FOXN3* | ENSG00000053254 | forkhead box N3 | 1928 |
| *FXR1* | ENSG00000114416 | FMR1 autosomal homolog 1 | 4023 |
| *GABRA4* | ENSG00000109158 | gamma-aminobutyric acid type A receptor subunit alpha4 | 4078 |
| *GAN* | ENSG00000261609 | gigaxonin | 4137 |
| *GLIPR1* | ENSG00000139278 | GLI pathogenesis related 1 | 17001 |
| *HECTD2* | ENSG00000165338 | HECT domain E3 ubiquitin protein ligase 2 | 26736 |
| *KCNN3* | ENSG00000143603 | potassium calcium-activated channel subfamily N member 3 | 6292 |
| *KCNS1* | ENSG00000124134 | potassium voltage-gated channel modifier subfamily S member 1 | 6300 |
| *KDM7A* | ENSG00000006459 | lysine demethylase 7A | 22224 |
| *L3MBTL3* | ENSG00000198945 | L3MBTL histone methyl-lysine binding protein 3 | 23035 |
| *LPP* | ENSG00000145012 | LIM domain containing preferred translocation partner in lipoma | 6679 |
| *MAN1A2* | ENSG00000198162 | mannosidase alpha class 1A member 2 | 6822 |
| *MECP2* | ENSG00000169057 | methyl-CpG binding protein 2 | 6990 |
| *METTL8* | ENSG00000123600 | methyltransferase 8, tRNA N3-cytidine | 25856 |
| *NETO1* | ENSG00000166342 | neuropilin and tolloid like 1 | 13823 |
| *NFAT5* | ENSG00000102908 | nuclear factor of activated T cells 5 | 7774 |
| *NR5A2* | ENSG00000116833 | nuclear receptor subfamily 5 group A member 2 | 7984 |
| *OTULINL* | ENSG00000145569 | OTU deubiquitinase with linear linkage specificity like | 25629 |
| *PAX5* | ENSG00000196092 | paired box 5 | 8619 |
| *PDE5A* | ENSG00000138735 | phosphodiesterase 5A | 8784 |
| *POU3F3* | ENSG00000198914 | POU class 3 homeobox 3 | 9216 |
| *PPM1L* | ENSG00000163590 | protein phosphatase, Mg2+/Mn2+ dependent 1L | 16381 |
| *PTPRF* | ENSG00000142949 | protein tyrosine phosphatase receptor type F | 9670 |
| *RBPJ* | ENSG00000168214 | recombination signal binding protein for immunoglobulin kappa J region | 5724 |
| *RC3H1* | ENSG00000135870 | ring finger and CCCH-type domains 1 | 29434 |
| *RCOR1* | ENSG00000089902 | REST corepressor 1 | 17441 |
| *RNGTT* | ENSG00000111880 | RNA guanylyltransferase and 5'-phosphatase | 10073 |
| *SEC62* | ENSG00000008952 | SEC62 homolog, preprotein translocation factor | 11846 |
| *SENP6* | ENSG00000112701 | SUMO specific peptidase 6 | 20944 |
| *SLC24A2* | ENSG00000155886 | solute carrier family 24 member 2 | 10976 |
| *SORT1* | ENSG00000134243 | sortilin 1 | 11186 |
| *SRGAP2* | ENSG00000266028 | SLIT-ROBO Rho GTPase activating protein 2 | 19751 |
| *SS18* | ENSG00000141380 | SS18 subunit of BAF chromatin remodeling complex | 11340 |
| *SV2B* | ENSG00000185518 | synaptic vesicle glycoprotein 2B | 16874 |
| *TET2* | ENSG00000168769 | tet methylcytosine dioxygenase 2 | 25941 |
| *TGFB2* | ENSG00000092969 | transforming growth factor beta 2 | 11768 |
| *TMEM108* | ENSG00000144868 | transmembrane protein 108 | 28451 |
| *TRABD2B* | ENSG00000269113 | TraB domain containing 2B | 44200 |
| *TRDMT1* | ENSG00000107614 | tRNA aspartic acid methyltransferase 1 | 2977 |
| *TRIP12* | ENSG00000153827 | thyroid hormone receptor interactor 12 | 12306 |
| *UBR3* | ENSG00000144357 | ubiquitin protein ligase E3 component n-recognin 3 | 30467 |
| *ZEB2* | ENSG00000169554 | zinc finger E-box binding homeobox 2 | 14881 |
| *ZNF507* | ENSG00000168813 | zinc finger protein 507 | 23783 |
| *ZNF644* | ENSG00000122482 | zinc finger protein 644 | 29222 |

**Supplementary Table 4.** List of miRNA target genes categorized as upregulated or downregulated based on both DEG analysis and mean expression in each GSE dataset.

| **tissue** | **dataset** | **logFC** | **AveExpr** | **t** | **P.Value** | **adj.P.Val** | **B** | **Gene** | **direction** | **significance** |
| --- | --- | --- | --- | --- | --- | --- | --- | --- | --- | --- |
| Foot | GSE68183 | -0.5971 | 8.3326 | -3.7503 | 0.0080 | 0.8019 | -3.0146 | *FAT4* | up | ** |
|  |  | -0.3867 | 9.6533 | -2.8398 | 0.0267 | 0.8019 | -3.5052 | *SPRED1* | up | * |
|  |  | -0.7341 | 14.1027 | -2.6802 | 0.0334 | 0.8048 | -3.6053 | *RAB30* | up | * |
|  |  | -0.2648 | 8.6247 | -2.6635 | 0.0342 | 0.8048 | -3.6160 | *CMTM4* | up | * |
|  |  | -0.5010 | 6.0696 | -2.5537 | 0.0399 | 0.8048 | -3.6873 | *SLC1A2* | up | * |
|  |  | -1.0002 | 7.1895 | -2.5172 | 0.0420 | 0.8048 | -3.7114 | *SEMA3D* | up | * |
|  |  | -0.3248 | 9.5371 | -2.4983 | 0.0432 | 0.8048 | -3.7240 | *TRIP11* | up | * |
|  |  | -0.4838 | 5.1140 | -2.3585 | 0.0527 | 0.8048 | -3.8183 | *WNK3* | up |  |
|  |  | -0.3268 | 6.7911 | -2.3161 | 0.0560 | 0.8048 | -3.8473 | *PGM2L1* | up |  |
|  |  | -0.2777 | 9.3399 | -2.2935 | 0.0578 | 0.8048 | -3.8629 | *ZCCHC2* | up |  |
|  |  | -0.4654 | 5.5780 | -2.1475 | 0.0713 | 0.8069 | -3.9647 | *LGI2* | up |  |
|  |  | -0.2133 | 9.0727 | -2.0412 | 0.0831 | 0.8073 | -4.0401 | *ROCK2* | up |  |
|  |  | -0.5271 | 8.8071 | -2.0380 | 0.0835 | 0.8073 | -4.0424 | *TRDN* | up |  |
|  |  | -0.3309 | 6.7472 | -2.0179 | 0.0860 | 0.8074 | -4.0567 | *SOBP* | up |  |
|  |  | -0.2081 | 9.0769 | -1.9912 | 0.0893 | 0.8078 | -4.0757 | *PEAK1* | up |  |
|  |  | 0.3427 | 14.0237 | 2.7574 | 0.0300 | 0.8048 | -3.5564 | *TET2* | down | * |
|  |  | 0.4010 | 7.5788 | 2.5487 | 0.0402 | 0.8048 | -3.6906 | *DENND1B* | down | * |
|  |  | 0.2687 | 10.9923 | 2.3452 | 0.0537 | 0.8048 | -3.8273 | *SORT1* | down |  |
|  |  | 0.3118 | 16.1453 | 2.1711 | 0.0690 | 0.8055 | -3.9482 | *EML4* | down |  |
|  |  | 0.3512 | 5.4022 | 1.9931 | 0.0891 | 0.8078 | -4.0743 | *GABRA4* | down |  |
|  | GSE80178 | -2.2314 | 8.3192 | -8.5250 | 0.0000 | 0.0007 | 4.1845 | *KMT2A* | up | *** |
|  |  | -1.8886 | 6.7210 | -8.0902 | 0.0000 | 0.0008 | 3.7263 | *CREBRF* | up | *** |
|  |  | -1.8143 | 7.1982 | -7.9577 | 0.0000 | 0.0009 | 3.5824 | *FAT4* | up | *** |
|  |  | -2.0825 | 6.8913 | -7.4378 | 0.0000 | 0.0012 | 2.9976 | *SEMA6D* | up | *** |
|  |  | -1.5957 | 11.3674 | -7.1748 | 0.0000 | 0.0014 | 2.6892 | *NF1* | up | *** |
|  |  | -2.0296 | 12.8197 | -6.8684 | 0.0000 | 0.0017 | 2.3186 | *RAB30* | up | *** |
|  |  | -2.4586 | 5.9638 | -6.8038 | 0.0001 | 0.0018 | 2.2389 | *SEMA3D* | up | *** |
|  |  | -1.2944 | 6.3517 | -6.7583 | 0.0001 | 0.0018 | 2.1824 | *PTCH1* | up | *** |
|  |  | -4.6306 | 26.8069 | -6.6968 | 0.0001 | 0.0019 | 2.1056 | *ZBTB20* | up | *** |
|  |  | -1.4262 | 6.9749 | -5.9892 | 0.0001 | 0.0032 | 1.1845 | *SREK1* | up | *** |
|  |  | -1.8748 | 6.5994 | -5.9562 | 0.0002 | 0.0033 | 1.1397 | *NTRK2* | up | *** |
|  |  | -1.2830 | 6.9033 | -5.9275 | 0.0002 | 0.0034 | 1.1007 | *PLXNC1* | up | *** |
|  |  | -1.3997 | 6.8250 | -5.6733 | 0.0002 | 0.0042 | 0.7501 | *ZC3H12C* | up | *** |
|  |  | -1.9998 | 8.1461 | -5.6619 | 0.0002 | 0.0042 | 0.7341 | *MDM4* | up | *** |
|  |  | -2.0364 | 7.8389 | -5.2612 | 0.0004 | 0.0059 | 0.1615 | *TNRC6B* | up | *** |
|  |  | -2.1460 | 7.8804 | -4.9547 | 0.0006 | 0.0076 | -0.2925 | *UBN2* | up | *** |
|  |  | -1.2270 | 7.4535 | -4.7530 | 0.0008 | 0.0090 | -0.5986 | *CMTM4* | up | *** |
|  |  | -1.7664 | 7.2311 | -4.7375 | 0.0009 | 0.0091 | -0.6224 | *CAAP1* | up | *** |
|  |  | -1.3912 | 6.5635 | -4.7178 | 0.0009 | 0.0093 | -0.6527 | *CHIC1* | up | *** |
|  |  | -1.2718 | 6.6036 | -4.4957 | 0.0012 | 0.0114 | -0.9975 | *ADAMTS5* | up | ** |
|  |  | -1.6614 | 8.1569 | -4.4354 | 0.0013 | 0.0120 | -1.0924 | *ZCCHC2* | up | ** |
|  |  | -0.7918 | 7.4550 | -4.3663 | 0.0015 | 0.0128 | -1.2015 | *TLE4* | up | ** |
|  |  | -1.2435 | 7.2207 | -4.3542 | 0.0015 | 0.0130 | -1.2207 | *ZNF362* | up | ** |
|  |  | -0.8525 | 6.3407 | -4.3102 | 0.0016 | 0.0136 | -1.2907 | *DIXDC1* | up | ** |
|  |  | -1.7869 | 8.2170 | -4.2301 | 0.0018 | 0.0146 | -1.4186 | *DMTF1* | up | ** |
|  |  | -2.1384 | 8.6983 | -4.0815 | 0.0023 | 0.0165 | -1.6580 | *CHD9* | up | ** |
|  |  | -1.1186 | 6.5907 | -4.0614 | 0.0024 | 0.0168 | -1.6908 | *CDC42SE2* | up | ** |
|  |  | -0.8347 | 5.8627 | -4.0426 | 0.0025 | 0.0171 | -1.7212 | *CACNB4* | up | ** |
|  |  | -0.9222 | 6.4988 | -3.9906 | 0.0027 | 0.0179 | -1.8059 | *CPEB3* | up | ** |
|  |  | -1.4233 | 8.6539 | -3.9402 | 0.0029 | 0.0189 | -1.8883 | *EZH1* | up | ** |
|  |  | -1.7846 | 7.3445 | -3.8679 | 0.0033 | 0.0202 | -2.0067 | *PTPN4* | up | ** |
|  |  | -1.2345 | 7.2643 | -3.8457 | 0.0034 | 0.0206 | -2.0433 | *GOLGA1* | up | ** |
|  |  | -1.7549 | 8.8519 | -3.7771 | 0.0038 | 0.0220 | -2.1564 | *INO80D* | up | ** |
|  |  | -0.8693 | 6.5902 | -3.7461 | 0.0040 | 0.0227 | -2.2078 | *ELK4* | up | ** |
|  |  | -2.0906 | 8.1522 | -3.6771 | 0.0044 | 0.0242 | -2.3221 | *SPRED1* | up | ** |
|  |  | -1.5735 | 9.1937 | -3.5490 | 0.0055 | 0.0276 | -2.5356 | *ANKIB1* | up | ** |
|  |  | -1.3214 | 7.8410 | -3.5222 | 0.0057 | 0.0284 | -2.5805 | *TP53INP1* | up | ** |
|  |  | -1.2409 | 7.8185 | -3.3980 | 0.0070 | 0.0322 | -2.7888 | *MBTPS2* | up | ** |
|  |  | -1.5672 | 7.8532 | -3.2959 | 0.0083 | 0.0358 | -2.9607 | *PEAK1* | up | ** |
|  |  | -1.2878 | 7.9263 | -3.2600 | 0.0089 | 0.0370 | -3.0212 | *FRS2* | up | ** |
|  |  | -1.1988 | 7.5583 | -3.1856 | 0.0100 | 0.0399 | -3.1468 | *PAG1* | up | * |
|  |  | -1.3369 | 7.6654 | -3.1854 | 0.0100 | 0.0399 | -3.1472 | *CPSF6* | up | * |
|  |  | -1.0921 | 7.5635 | -3.1583 | 0.0105 | 0.0410 | -3.1930 | *OTUD4* | up | * |
|  |  | -0.5878 | 6.6118 | -3.1454 | 0.0107 | 0.0416 | -3.2146 | *ZBTB18* | up | * |
|  |  | -0.7671 | 5.6505 | -3.1301 | 0.0110 | 0.0422 | -3.2406 | *ADAMTS3* | up | * |
|  |  | -1.1959 | 8.3457 | -3.1233 | 0.0111 | 0.0426 | -3.2520 | *CNOT6L* | up | * |
|  |  | -1.5472 | 8.6792 | -3.1163 | 0.0113 | 0.0429 | -3.2639 | *SNTB2* | up | * |
|  |  | -1.1031 | 7.8098 | -3.0667 | 0.0122 | 0.0453 | -3.3478 | *ARMC8* | up | * |
|  |  | -1.4935 | 7.5275 | -3.0556 | 0.0125 | 0.0458 | -3.3664 | *SLC25A36* | up | * |
|  |  | -1.3790 | 7.6439 | -2.8782 | 0.0168 | 0.0554 | -3.6661 | *DPP8* | up | * |
|  |  | -0.6752 | 7.6574 | -2.8479 | 0.0177 | 0.0572 | -3.7172 | *MAPK9* | up | * |
|  |  | -0.6248 | 6.5384 | -2.8283 | 0.0183 | 0.0584 | -3.7501 | *SOBP* | up | * |
|  |  | -1.2419 | 8.2302 | -2.7838 | 0.0198 | 0.0613 | -3.8251 | *USP31* | up | * |
|  |  | -1.8019 | 8.2540 | -2.7709 | 0.0202 | 0.0621 | -3.8468 | *TRIP11* | up | * |
|  |  | -1.6427 | 7.9858 | -2.7112 | 0.0223 | 0.0665 | -3.9471 | *RICTOR* | up | * |
|  |  | -1.5993 | 7.7915 | -2.6652 | 0.0242 | 0.0699 | -4.0241 | *ROCK2* | up | * |
|  |  | -1.5007 | 7.5940 | -2.6645 | 0.0242 | 0.0700 | -4.0253 | *PTEN* | up | * |
|  |  | -1.2573 | 7.3418 | -2.6234 | 0.0259 | 0.0732 | -4.0938 | *GXYLT1* | up | * |
|  |  | -0.9013 | 8.8320 | -2.5937 | 0.0273 | 0.0756 | -4.1435 | *SETD3* | up | * |
|  |  | -0.6450 | 7.3664 | -2.5376 | 0.0300 | 0.0805 | -4.2366 | *SEC22C* | up | * |
|  |  | -0.5163 | 8.0451 | -2.5276 | 0.0305 | 0.0813 | -4.2533 | *ITPRIPL2* | up | * |
|  |  | -0.6089 | 7.1421 | -2.5207 | 0.0309 | 0.0819 | -4.2648 | *STOX2* | up | * |
|  |  | -0.6088 | 8.0139 | -2.4910 | 0.0325 | 0.0845 | -4.3139 | *QKI* | up | * |
|  |  | -1.3636 | 8.6232 | -2.3325 | 0.0425 | 0.1008 | -4.5739 | *PDK3* | up | * |
|  |  | -1.3372 | 9.3580 | -2.2463 | 0.0491 | 0.1113 | -4.7134 | *GOLGA4* | up | * |
|  |  | -0.6213 | 6.5406 | -2.1854 | 0.0544 | 0.1191 | -4.8110 | *SLC39A10* | up |  |
|  |  | -0.5951 | 4.6742 | -2.1244 | 0.0603 | 0.1279 | -4.9079 | *WNK3* | up |  |
|  |  | -0.9696 | 13.7103 | -2.0811 | 0.0648 | 0.1343 | -4.9760 | *CCNT2* | up |  |
|  |  | -0.5697 | 8.2522 | -2.0196 | 0.0717 | 0.1441 | -5.0718 | *SINHCAF* | up |  |
|  |  | -0.5322 | 14.1944 | -2.0006 | 0.0740 | 0.1474 | -5.1013 | *RNF213* | up |  |
|  |  | -0.7362 | 7.1983 | -1.9555 | 0.0797 | 0.1554 | -5.1704 | *CDC37L1* | up |  |
|  |  | -0.6100 | 18.5389 | -1.9496 | 0.0805 | 0.1564 | -5.1794 | *SMAD1* | up |  |
|  |  | -1.0225 | 7.4223 | -1.9214 | 0.0843 | 0.1618 | -5.2224 | *BCL11B* | up |  |
|  |  | -0.9193 | 8.6469 | -1.8938 | 0.0883 | 0.1667 | -5.2642 | *FNDC3B* | up |  |
|  |  | -1.1741 | 8.0520 | -1.8901 | 0.0888 | 0.1673 | -5.2698 | *WEE1* | up |  |
|  |  | -0.9286 | 9.7394 | -1.8733 | 0.0913 | 0.1704 | -5.2950 | *SSR1* | up |  |
|  |  | -0.6646 | 9.0660 | -1.8516 | 0.0945 | 0.1748 | -5.3274 | *DAZAP2* | up |  |
|  |  | -0.6572 | 7.6724 | -1.8439 | 0.0957 | 0.1761 | -5.3389 | *PPM1A* | up |  |
|  |  | -1.0874 | 5.9147 | -1.8377 | 0.0967 | 0.1774 | -5.3481 | *EIF5A2* | up |  |
|  |  | -0.7560 | 8.2577 | -1.8172 | 0.1000 | 0.1818 | -5.3785 | *RNF111* | up |  |
|  |  | 0.8766 | 6.3489 | 4.9034 | 0.0007 | 0.0079 | -0.3698 | *PAX5* | down | *** |
|  |  | 1.0476 | 6.1399 | 4.0876 | 0.0023 | 0.0164 | -1.6481 | *KCNS1* | down | ** |
|  |  | 1.0286 | 15.1337 | 3.0970 | 0.0116 | 0.0437 | -3.2966 | *POU3F3* | down | * |
|  |  | 0.6222 | 5.1445 | 2.8625 | 0.0173 | 0.0563 | -3.6926 | *GABRA4* | down | * |
|  |  | 1.2752 | 9.1248 | 2.7323 | 0.0216 | 0.0649 | -3.9116 | *GLIPR1* | down | * |
|  |  | 0.8264 | 5.2278 | 2.7063 | 0.0225 | 0.0669 | -3.9552 | *NETO1* | down | * |
|  |  | 0.4497 | 5.7120 | 2.6430 | 0.0251 | 0.0716 | -4.0612 | *AMER2* | down | * |
|  |  | 0.6992 | 4.2335 | 2.6206 | 0.0261 | 0.0734 | -4.0986 | *SLC24A2* | down | * |
|  |  | 0.4597 | 10.9235 | 2.1743 | 0.0554 | 0.1205 | -4.8287 | *SORT1* | down |  |
|  |  | 2.3898 | 17.5042 | 2.0955 | 0.0632 | 0.1322 | -4.9534 | *KDM7A* | down |  |
|  |  | 0.8228 | 7.8026 | 1.8450 | 0.0956 | 0.1759 | -5.3372 | *DENND1B* | down |  |
|  | GSE199939 | -25.2242 | 22.9467 | -6.4592 | 0.0000 | 0.0001 | 3.2334 | *EZH1* | up | *** |
|  |  | -0.8879 | 1.0081 | -6.3368 | 0.0000 | 0.0001 | 2.9700 | *CPEB3* | up | *** |
|  |  | -10.4843 | 8.6148 | -6.2327 | 0.0000 | 0.0002 | 2.7448 | *RGMA* | up | *** |
|  |  | -1.6681 | 1.3448 | -5.6397 | 0.0000 | 0.0004 | 1.4378 | *STOX2* | up | *** |
|  |  | -2.0686 | 2.5886 | -5.6166 | 0.0000 | 0.0005 | 1.3862 | *UBN2* | up | *** |
|  |  | -2.0709 | 2.8648 | -5.2629 | 0.0000 | 0.0008 | 0.5891 | *INO80D* | up | *** |
|  |  | -12.9245 | 28.0210 | -5.2585 | 0.0000 | 0.0008 | 0.5790 | *YTHDC1* | up | *** |
|  |  | -1.1912 | 1.9410 | -5.0608 | 0.0001 | 0.0012 | 0.1290 | *POU2F1* | up | *** |
|  |  | -5.9347 | 8.3567 | -4.9812 | 0.0001 | 0.0013 | -0.0527 | *MDM4* | up | *** |
|  |  | -2.8241 | 7.8143 | -4.9585 | 0.0001 | 0.0014 | -0.1048 | *GOLGA1* | up | *** |
|  |  | -2.7339 | 1.8390 | -4.7245 | 0.0001 | 0.0021 | -0.6417 | *SEMA3D* | up | *** |
|  |  | -3.1871 | 5.5714 | -4.6888 | 0.0002 | 0.0022 | -0.7240 | *BRWD1* | up | *** |
|  |  | -1.4168 | 1.0271 | -4.5574 | 0.0002 | 0.0028 | -1.0267 | *CACNB4* | up | *** |
|  |  | -17.0175 | 25.2319 | -4.5238 | 0.0002 | 0.0030 | -1.1041 | *DMTF1* | up | *** |
|  |  | -6.2640 | 21.8271 | -4.4304 | 0.0003 | 0.0035 | -1.3194 | *TBL1XR1* | up | *** |
|  |  | -4.9692 | 15.3319 | -4.3228 | 0.0004 | 0.0042 | -1.5677 | *WASL* | up | *** |
|  |  | -3.4961 | 9.3243 | -4.1631 | 0.0005 | 0.0056 | -1.9358 | *DLGAP1* | up | *** |
|  |  | -6.0646 | 12.5257 | -4.1430 | 0.0005 | 0.0058 | -1.9820 | *RAB30* | up | *** |
|  |  | -32.7416 | 18.4724 | -4.0301 | 0.0007 | 0.0070 | -2.2418 | *NTRK2* | up | *** |
|  |  | -6.3191 | 13.1500 | -4.0281 | 0.0007 | 0.0071 | -2.2463 | *MTMR3* | up | *** |
|  |  | -4.2916 | 2.6700 | -3.9504 | 0.0008 | 0.0081 | -2.4248 | *MYO5B* | up | *** |
|  |  | -1.5121 | 1.5290 | -3.8898 | 0.0010 | 0.0090 | -2.5635 | *ZBTB20* | up | *** |
|  |  | -23.7359 | 79.7081 | -3.8679 | 0.0010 | 0.0094 | -2.6137 | *SON* | up | ** |
|  |  | -3.2990 | 13.4090 | -3.8265 | 0.0011 | 0.0101 | -2.7083 | *ZNF362* | up | ** |
|  |  | -3.3694 | 7.0119 | -3.7859 | 0.0012 | 0.0109 | -2.8013 | *ZCCHC2* | up | ** |
|  |  | -1.4045 | 1.0057 | -3.7292 | 0.0014 | 0.0120 | -2.9303 | *SLC1A2* | up | ** |
|  |  | -2.7239 | 4.5238 | -3.5125 | 0.0023 | 0.0176 | -3.4211 | *CREBRF* | up | ** |
|  |  | -1.9527 | 3.1929 | -3.4744 | 0.0025 | 0.0188 | -3.5067 | *PTCH1* | up | ** |
|  |  | -5.5236 | 14.0233 | -3.4657 | 0.0025 | 0.0191 | -3.5263 | *SLC25A36* | up | ** |
|  |  | -6.7177 | 9.0138 | -3.3281 | 0.0035 | 0.0242 | -3.8335 | *CPEB2* | up | ** |
|  |  | -4.0338 | 9.4910 | -3.2734 | 0.0039 | 0.0264 | -3.9547 | *KMT2A* | up | ** |
|  |  | -14.1185 | 24.5614 | -3.2473 | 0.0042 | 0.0277 | -4.0124 | *MAFK* | up | ** |
|  |  | -1.9292 | 2.4595 | -3.2411 | 0.0042 | 0.0280 | -4.0261 | *SEMA6D* | up | ** |
|  |  | -3.2739 | 8.2119 | -3.1762 | 0.0049 | 0.0313 | -4.1687 | *TP53INP1* | up | ** |
|  |  | -4.9983 | 13.4671 | -3.0404 | 0.0067 | 0.0393 | -4.4641 | *GOLGA4* | up | ** |
|  |  | -4.6530 | 16.1343 | -3.0177 | 0.0070 | 0.0408 | -4.5132 | *ARMC8* | up | ** |
|  |  | -0.9172 | 1.9614 | -2.9955 | 0.0074 | 0.0423 | -4.5610 | *CHIC1* | up | ** |
|  |  | -1.4753 | 1.2948 | -2.9697 | 0.0078 | 0.0442 | -4.6163 | *RET* | up | ** |
|  |  | -0.7283 | 0.4705 | -2.9495 | 0.0081 | 0.0457 | -4.6595 | *MAP3K9* | up | ** |
|  |  | -21.7921 | 15.9319 | -2.8814 | 0.0095 | 0.0510 | -4.8043 | *RORA* | up | ** |
|  |  | -2.3736 | 10.2933 | -2.8338 | 0.0105 | 0.0553 | -4.9048 | *ANKIB1* | up | * |
|  |  | -3.6699 | 10.3133 | -2.8161 | 0.0109 | 0.0570 | -4.9419 | *CCNT2* | up | * |
|  |  | -1.8263 | 7.2476 | -2.7553 | 0.0125 | 0.0628 | -5.0688 | *CAAP1* | up | * |
|  |  | -9.9232 | 21.1829 | -2.7394 | 0.0129 | 0.0645 | -5.1018 | *HIPK3* | up | * |
|  |  | -0.0903 | 0.1143 | -2.7389 | 0.0129 | 0.0645 | -5.1028 | *SCN2A* | up | * |
|  |  | -1.3362 | 5.0719 | -2.6196 | 0.0167 | 0.0785 | -5.3476 | *FAM199X* | up | * |
|  |  | -1.8541 | 4.2662 | -2.5996 | 0.0175 | 0.0810 | -5.3881 | *SNX16* | up | * |
|  |  | -2.0050 | 4.1852 | -2.5941 | 0.0177 | 0.0818 | -5.3992 | *RICTOR* | up | * |
|  |  | -2.3798 | 4.6386 | -2.5779 | 0.0183 | 0.0839 | -5.4320 | *RNF217* | up | * |
|  |  | -1.9535 | 1.6343 | -2.5301 | 0.0203 | 0.0906 | -5.5279 | *BCL11B* | up | * |
|  |  | -1.7635 | 8.3457 | -2.5137 | 0.0210 | 0.0930 | -5.5606 | *DENND6A* | up | * |
|  |  | -1.8605 | 6.2986 | -2.4924 | 0.0220 | 0.0961 | -5.6029 | *ELK4* | up | * |
|  |  | -8.3229 | 9.8976 | -2.4787 | 0.0226 | 0.0982 | -5.6300 | *RASGEF1B* | up | * |
|  |  | -3.3848 | 12.9800 | -2.4661 | 0.0232 | 0.1001 | -5.6547 | *CHD9* | up | * |
|  |  | -3.9573 | 3.8829 | -2.3937 | 0.0270 | 0.1124 | -5.7964 | *YOD1* | up | * |
|  |  | -1.2009 | 4.7590 | -2.3360 | 0.0304 | 0.1234 | -5.9076 | *TAB3* | up | * |
|  |  | -1.3850 | 3.2405 | -2.3192 | 0.0315 | 0.1266 | -5.9395 | *PTPN4* | up | * |
|  |  | -0.4544 | 0.4400 | -2.2827 | 0.0340 | 0.1342 | -6.0088 | *PLAG1* | up | * |
|  |  | -23.9084 | 20.3414 | -2.2825 | 0.0340 | 0.1343 | -6.0093 | *IFFO2* | up | * |
|  |  | -0.5827 | 2.3252 | -2.2420 | 0.0369 | 0.1428 | -6.0853 | *ZDHHC21* | up | * |
|  |  | -1.5483 | 5.4400 | -2.2319 | 0.0377 | 0.1451 | -6.1043 | *FRS2* | up | * |
|  |  | -0.2904 | 0.3881 | -2.2315 | 0.0377 | 0.1451 | -6.1049 | *EPHA7* | up | * |
|  |  | -84.7853 | 97.1733 | -2.1893 | 0.0411 | 0.1547 | -6.1832 | *BTG2* | up | * |
|  |  | -0.5807 | 1.4662 | -2.1780 | 0.0420 | 0.1575 | -6.2040 | *ACVR2B* | up | * |
|  |  | -3.9487 | 13.2624 | -2.1342 | 0.0459 | 0.1682 | -6.2840 | *CDC37L1* | up | * |
|  |  | -3.9979 | 15.4271 | -2.0876 | 0.0503 | 0.1796 | -6.3679 | *RAP2C* | up |  |
|  |  | -13.1692 | 15.0371 | -2.0824 | 0.0509 | 0.1811 | -6.3773 | *WEE1* | up |  |
|  |  | -1.7827 | 6.7038 | -2.0522 | 0.0540 | 0.1890 | -6.4310 | *ZBTB18* | up |  |
|  |  | -0.0570 | 0.0729 | -2.0501 | 0.0542 | 0.1895 | -6.4347 | *UNC80* | up |  |
|  |  | -0.1845 | 0.1667 | -1.9835 | 0.0618 | 0.2092 | -6.5513 | *SCN1A* | up |  |
|  |  | -0.5462 | 2.0681 | -1.8928 | 0.0735 | 0.2380 | -6.7059 | *ZC3H12C* | up |  |
|  |  | -0.6105 | 0.6138 | -1.8450 | 0.0805 | 0.2537 | -6.7854 | *SLC4A4* | up |  |
|  |  | -1.7602 | 2.5500 | -1.8133 | 0.0854 | 0.2646 | -6.8373 | *ZFHX4* | up |  |
|  |  | -4.0351 | 26.3776 | -1.8093 | 0.0860 | 0.2660 | -6.8440 | *SETD3* | up |  |
|  |  | -2.4533 | 11.6290 | -1.7924 | 0.0888 | 0.2721 | -6.8713 | *ATXN1L* | up |  |
|  |  | -5.3424 | 17.0824 | -1.7710 | 0.0924 | 0.2803 | -6.9057 | *SREK1* | up |  |
|  |  | 42.8608 | 33.9281 | 7.4052 | 0.0000 | 0.0000 | 5.2041 | *GLIPR1* | down | *** |
|  |  | 21.9497 | 65.1895 | 6.5839 | 0.0000 | 0.0001 | 3.4997 | *CAPRIN1* | down | *** |
|  |  | 23.7215 | 37.1314 | 5.2998 | 0.0000 | 0.0008 | 0.6728 | *ENTPD1* | down | *** |
|  |  | 1.1699 | 3.5962 | 5.0815 | 0.0001 | 0.0011 | 0.1762 | *RNGTT* | down | *** |
|  |  | 102.7099 | 231.6886 | 3.3119 | 0.0036 | 0.0248 | -3.8696 | *APLP2* | down | ** |
|  |  | 1.2450 | 3.9729 | 2.6131 | 0.0170 | 0.0793 | -5.3608 | *L3MBTL3* | down | * |
|  |  | 4.2551 | 16.0671 | 2.1811 | 0.0418 | 0.1567 | -6.1983 | *FECH* | down | * |
|  |  | 0.6437 | 2.0038 | 2.0569 | 0.0535 | 0.1877 | -6.4227 | *DGKH* | down |  |
|  |  | 0.8881 | 2.4038 | 2.0228 | 0.0572 | 0.1970 | -6.4827 | *TRDMT1* | down |  |
|  |  | 28.9788 | 26.0276 | 1.8624 | 0.0779 | 0.2480 | -6.7566 | *EDIL3* | down |  |
| Kidney | GSE142025 | -2.2931 | 12.3091 | -10.0612 | 0.0000 | 0.0001 | 8.8240 | *BTG2* | up | *** |
|  |  | -0.5178 | 9.7210 | -8.6424 | 0.0000 | 0.0002 | 7.0132 | *CHIC1* | up | *** |
|  |  | -0.3966 | 11.7447 | -6.6724 | 0.0000 | 0.0015 | 4.0401 | *SLC25A36* | up | *** |
|  |  | -0.3697 | 11.6570 | -6.6305 | 0.0000 | 0.0016 | 3.9706 | *ETNK1* | up | *** |
|  |  | -0.3489 | 12.1499 | -5.3741 | 0.0001 | 0.0042 | 1.7640 | *WASL* | up | *** |
|  |  | -0.3096 | 8.4481 | -5.1569 | 0.0001 | 0.0052 | 1.3597 | *EIF5A2* | up | *** |
|  |  | -0.5382 | 7.7815 | -4.9382 | 0.0002 | 0.0062 | 0.9467 | *SNX16* | up | *** |
|  |  | -0.7022 | 9.8109 | -4.6486 | 0.0003 | 0.0079 | 0.3911 | *WEE1* | up | *** |
|  |  | -0.3769 | 10.0876 | -4.6409 | 0.0003 | 0.0079 | 0.3762 | *GXYLT1* | up | *** |
|  |  | -0.3058 | 10.5432 | -4.4704 | 0.0004 | 0.0090 | 0.0450 | *TLE4* | up | *** |
|  |  | -0.6335 | 10.8169 | -4.1289 | 0.0009 | 0.0124 | -0.6252 | *MAFK* | up | *** |
|  |  | -0.2538 | 10.4455 | -4.1053 | 0.0009 | 0.0127 | -0.6718 | *REEP3* | up | *** |
|  |  | -0.6075 | 12.9868 | -4.1026 | 0.0009 | 0.0128 | -0.6773 | *GOLGA4* | up | *** |
|  |  | -0.3110 | 11.8504 | -4.0972 | 0.0009 | 0.0129 | -0.6879 | *RAB10* | up | *** |
|  |  | -0.3379 | 10.3279 | -4.0912 | 0.0010 | 0.0129 | -0.6998 | *KPNA3* | up | *** |
|  |  | -0.1986 | 13.1315 | -3.8044 | 0.0017 | 0.0174 | -1.2678 | *DAZAP2* | up | ** |
|  |  | -0.2346 | 11.9221 | -3.6328 | 0.0024 | 0.0210 | -1.6083 | *ADAM10* | up | ** |
|  |  | -0.2431 | 10.3857 | -3.5854 | 0.0027 | 0.0221 | -1.7024 | *ZDHHC21* | up | ** |
|  |  | -0.2103 | 10.3087 | -3.5620 | 0.0028 | 0.0227 | -1.7488 | *MBTPS2* | up | ** |
|  |  | -0.3021 | 10.2944 | -3.5156 | 0.0031 | 0.0238 | -1.8406 | *DCUN1D1* | up | ** |
|  |  | -0.3450 | 11.2090 | -3.4774 | 0.0034 | 0.0250 | -1.9164 | *SREK1* | up | ** |
|  |  | -0.2151 | 9.5331 | -3.4490 | 0.0036 | 0.0258 | -1.9726 | *RNF217* | up | ** |
|  |  | -0.2159 | 11.2314 | -3.3963 | 0.0040 | 0.0276 | -2.0767 | *CDC42SE2* | up | ** |
|  |  | -0.1555 | 11.6306 | -3.3294 | 0.0045 | 0.0300 | -2.2087 | *FAM91A1* | up | ** |
|  |  | -1.7102 | 44.5369 | -3.3025 | 0.0048 | 0.0309 | -2.2616 | *ZBTB20* | up | ** |
|  |  | -0.2074 | 11.6363 | -3.2371 | 0.0055 | 0.0334 | -2.3902 | *CPSF6* | up | ** |
|  |  | -0.2559 | 11.8399 | -3.2058 | 0.0059 | 0.0348 | -2.4515 | *RICTOR* | up | ** |
|  |  | -0.3273 | 12.1146 | -3.1458 | 0.0066 | 0.0376 | -2.5690 | *ROCK2* | up | ** |
|  |  | -0.4045 | 9.3414 | -3.1122 | 0.0071 | 0.0392 | -2.6346 | *CACNB4* | up | ** |
|  |  | -0.4661 | 11.4464 | -3.0355 | 0.0083 | 0.0431 | -2.7838 | *CREBRF* | up | ** |
|  |  | -0.3796 | 15.1696 | -2.9828 | 0.0092 | 0.0462 | -2.8859 | *APP* | up | ** |
|  |  | -0.1706 | 12.0963 | -2.9438 | 0.0100 | 0.0488 | -2.9611 | *TBL1XR1* | up | * |
|  |  | -0.3422 | 8.6660 | -2.9305 | 0.0103 | 0.0496 | -2.9867 | *PGM2L1* | up | * |
|  |  | -0.4395 | 9.3099 | -2.8870 | 0.0112 | 0.0525 | -3.0704 | *RASGEF1B* | up | * |
|  |  | -0.1673 | 10.0346 | -2.8203 | 0.0129 | 0.0572 | -3.1978 | *CARNMT1* | up | * |
|  |  | -0.1499 | 12.1375 | -2.8084 | 0.0132 | 0.0580 | -3.2204 | *QKI* | up | * |
|  |  | -0.1720 | 10.6831 | -2.7661 | 0.0144 | 0.0611 | -3.3007 | *DENND6A* | up | * |
|  |  | -0.3403 | 11.4917 | -2.7121 | 0.0160 | 0.0661 | -3.4026 | *TP53INP1* | up | * |
|  |  | -0.1829 | 10.3353 | -2.6940 | 0.0166 | 0.0678 | -3.4367 | *SLC39A10* | up | * |
|  |  | -0.2089 | 11.6217 | -2.6930 | 0.0166 | 0.0679 | -3.4386 | *CNOT6L* | up | * |
|  |  | -0.1709 | 11.4769 | -2.6568 | 0.0179 | 0.0716 | -3.5063 | *FAM199X* | up | * |
|  |  | -0.1432 | 11.7736 | -2.6212 | 0.0192 | 0.0752 | -3.5727 | *RLIM* | up | * |
|  |  | -0.2034 | 14.6495 | -2.4775 | 0.0255 | 0.0901 | -3.8372 | *AK4* | up | * |
|  |  | -0.3028 | 11.9869 | -2.4585 | 0.0265 | 0.0921 | -3.8719 | *ACSL4* | up | * |
|  |  | -0.1897 | 11.0933 | -2.4387 | 0.0276 | 0.0944 | -3.9077 | *SHOC2* | up | * |
|  |  | -0.1458 | 11.2873 | -2.4342 | 0.0278 | 0.0951 | -3.9159 | *GLCE* | up | * |
|  |  | -0.4618 | 9.3782 | -2.4297 | 0.0281 | 0.0956 | -3.9240 | *PCDH17* | up | * |
|  |  | -0.4473 | 17.8812 | -2.4222 | 0.0285 | 0.0965 | -3.9375 | *TAB3* | up | * |
|  |  | -0.6454 | 21.3273 | -2.4133 | 0.0290 | 0.0977 | -3.9536 | *RORA* | up | * |
|  |  | -0.2575 | 11.8104 | -2.4119 | 0.0291 | 0.0978 | -3.9561 | *TRIP11* | up | * |
|  |  | -0.1269 | 12.8582 | -2.3732 | 0.0313 | 0.1030 | -4.0255 | *PAFAH1B1* | up | * |
|  |  | -0.2310 | 9.6943 | -2.2491 | 0.0399 | 0.1215 | -4.2449 | *CDC37L1* | up | * |
|  |  | -0.1184 | 11.7722 | -2.2216 | 0.0420 | 0.1260 | -4.2927 | *SH3GLB1* | up | * |
|  |  | -0.1823 | 11.0938 | -2.1534 | 0.0479 | 0.1376 | -4.4100 | *SEC24A* | up | * |
|  |  | -0.3783 | 10.9639 | -2.1261 | 0.0504 | 0.1422 | -4.4564 | *ELL2* | up |  |
|  |  | -0.1636 | 11.2352 | -2.0608 | 0.0570 | 0.1552 | -4.5663 | *SRP72* | up |  |
|  |  | -1.4987 | 21.2365 | -2.0521 | 0.0579 | 0.1568 | -4.5808 | *DLGAP1* | up |  |
|  |  | -0.1565 | 10.4141 | -2.0497 | 0.0582 | 0.1572 | -4.5848 | *MEX3C* | up |  |
|  |  | -0.1395 | 11.6112 | -2.0024 | 0.0636 | 0.1668 | -4.6629 | *KPNA1* | up |  |
|  |  | -0.1603 | 10.7091 | -2.0021 | 0.0636 | 0.1668 | -4.6633 | *CPEB3* | up |  |
|  |  | -0.2848 | 11.6537 | -2.0017 | 0.0636 | 0.1669 | -4.6641 | *SKIL* | up |  |
|  |  | -0.1398 | 9.4921 | -1.9601 | 0.0687 | 0.1761 | -4.7318 | *CAAP1* | up |  |
|  |  | -0.2891 | 10.0573 | -1.9492 | 0.0701 | 0.1783 | -4.7495 | *YOD1* | up |  |
|  |  | -0.1316 | 10.9361 | -1.8902 | 0.0781 | 0.1921 | -4.8440 | *SPRED1* | up |  |
|  |  | -0.1419 | 12.7498 | -1.8671 | 0.0814 | 0.1974 | -4.8804 | *PDK3* | up |  |
|  |  | -0.1503 | 13.1992 | -1.7851 | 0.0944 | 0.2179 | -5.0078 | *CHD9* | up |  |
|  |  | 0.3329 | 11.0661 | 4.3843 | 0.0005 | 0.0098 | -0.1232 | *ZNF507* | down | *** |
|  |  | 0.2286 | 12.8318 | 4.2173 | 0.0007 | 0.0114 | -0.4510 | *MECP2* | down | *** |
|  |  | 0.4922 | 8.6917 | 3.2473 | 0.0054 | 0.0330 | -2.3702 | *ADGRL3* | down | ** |
|  |  | 0.7112 | 4.9129 | 3.1321 | 0.0068 | 0.0383 | -2.5959 | *KCNS1* | down | ** |
|  |  | 0.1957 | 10.2563 | 1.8216 | 0.0884 | 0.2088 | -4.9515 | *KCNN3* | down |  |
|  |  | 0.1845 | 12.0839 | 1.7730 | 0.0964 | 0.2210 | -5.0262 | *ZEB2* | down |  |
|  | GSE162830 | -526.9688 | 462.7743 | -5.5026 | 0.0000 | 0.0061 | -3.8922 | *MTMR3* | up | *** |
|  |  | -1044.2639 | 650.3831 | -5.3847 | 0.0000 | 0.0067 | -3.9077 | *GOLGA4* | up | *** |
|  |  | -926.9948 | 684.7917 | -4.4421 | 0.0001 | 0.0294 | -4.0447 | *PEAK1* | up | *** |
|  |  | -555.3056 | 509.9873 | -3.4787 | 0.0018 | 0.1386 | -4.2062 | *UBN2* | up | ** |
|  |  | -165.0503 | 179.9630 | -3.1229 | 0.0044 | 0.2226 | -4.2694 | *LATS2* | up | ** |
|  |  | -84.1233 | 118.7963 | -2.7992 | 0.0096 | 0.3469 | -4.3272 | *RNF111* | up | ** |
|  |  | -1050.5451 | 1293.7060 | -2.7914 | 0.0098 | 0.3513 | -4.3286 | *HIPK2* | up | ** |
|  |  | -1145.6215 | 1293.0544 | -2.7402 | 0.0110 | 0.3703 | -4.3377 | *KMT2A* | up | * |
|  |  | -844.1910 | 1129.8310 | -2.3734 | 0.0254 | 0.5431 | -4.4021 | *SON* | up | * |
|  |  | -139.9792 | 230.8993 | -2.2488 | 0.0333 | 0.6173 | -4.4233 | *SLC4A4* | up | * |
|  |  | -98.1007 | 127.9086 | -2.2204 | 0.0354 | 0.6320 | -4.4281 | *CREBRF* | up | * |
|  |  | -1908.0573 | 2853.6667 | -2.2130 | 0.0360 | 0.6365 | -4.4294 | *RNF213* | up | * |
|  |  | -332.3021 | 466.2639 | -2.1178 | 0.0440 | 0.7047 | -4.4452 | *FAT4* | up | * |
|  |  | -229.7795 | 269.8449 | -2.1150 | 0.0443 | 0.7051 | -4.4457 | *PAG1* | up | * |
|  |  | -263.0347 | 340.8900 | -2.0869 | 0.0470 | 0.7285 | -4.4503 | *EZH1* | up | * |
|  |  | -114.2795 | 161.1157 | -2.0290 | 0.0529 | 0.7654 | -4.4597 | *SEMA6D* | up |  |
|  |  | -722.1337 | 1042.5984 | -2.0045 | 0.0557 | 0.7818 | -4.4637 | *TNRC6B* | up |  |
|  |  | -331.7986 | 680.1065 | -1.9229 | 0.0656 | 0.8417 | -4.4767 | *BRWD1* | up |  |
|  |  | -131.2604 | 162.2917 | -1.8735 | 0.0724 | 0.8738 | -4.4844 | *SETD3* | up |  |
|  |  | -292.9028 | 681.2350 | -1.7995 | 0.0837 | 0.8825 | -4.4958 | *ZBTB20* | up |  |
|  |  | -179.7240 | 305.4201 | -1.7595 | 0.0904 | 0.8825 | -4.5018 | *TRIP11* | up |  |
|  |  | 75.8976 | 101.9387 | 2.1555 | 0.0407 | 0.6799 | -4.4390 | *POU3F3* | down | * |
|  |  | 85.4844 | 99.4792 | 1.8957 | 0.0693 | 0.8601 | -4.4809 | *RBPJ* | down |  |
|  |  | 69.1476 | 173.5567 | 1.8756 | 0.0721 | 0.8738 | -4.4841 | *CDK19* | down |  |
|  | GSE163603 | -1.7350 | 7.0066 | -2.5165 | 0.0248 | 0.3246 | -3.2747 | *YTHDC1* | up | * |
|  |  | 1.2776 | 7.9334 | 3.1976 | 0.0065 | 0.3017 | -2.1685 | *APC* | down | ** |
|  |  | 0.8910 | 8.3314 | 2.8574 | 0.0127 | 0.3146 | -2.7260 | *KCNN3* | down | * |
|  |  | 1.0307 | 8.2071 | 2.6095 | 0.0207 | 0.3245 | -3.1265 | *SLC24A2* | down | * |
|  |  | 1.3800 | 22.7950 | 2.6064 | 0.0208 | 0.3245 | -3.1314 | *LPP* | down | * |
|  |  | 1.0070 | 7.2772 | 2.5287 | 0.0242 | 0.3245 | -3.2553 | *CNOT2* | down | * |
|  |  | 0.5406 | 6.4093 | 2.4938 | 0.0259 | 0.3247 | -3.3105 | *GLIPR1* | down | * |
|  |  | 0.9773 | 14.6667 | 2.3527 | 0.0339 | 0.3279 | -3.5317 | *SRGAP2* | down | * |
|  |  | 0.6948 | 14.0823 | 2.2998 | 0.0375 | 0.3283 | -3.6134 | *HECTD2* | down | * |
|  |  | 0.8194 | 8.1432 | 2.2430 | 0.0417 | 0.3291 | -3.7004 | *KDM7A* | down | * |
|  |  | 0.9700 | 7.9867 | 2.1017 | 0.0543 | 0.3379 | -3.9129 | *RC3H1* | down |  |
|  |  | 0.5499 | 8.2786 | 2.0698 | 0.0576 | 0.3382 | -3.9600 | *FAM227A* | down |  |
|  |  | 0.5354 | 7.8058 | 2.0241 | 0.0626 | 0.3409 | -4.0269 | *PAX5* | down |  |
|  |  | 0.6304 | 7.6053 | 1.9535 | 0.0712 | 0.3457 | -4.1288 | *FECH* | down |  |
|  |  | 1.2280 | 8.8615 | 1.8812 | 0.0811 | 0.3519 | -4.2312 | *TRIP12* | down |  |
|  |  | 0.9231 | 7.2130 | 1.8320 | 0.0885 | 0.3569 | -4.2998 | *RBPJ* | down |  |
|  |  | 0.9920 | 8.1245 | 1.8287 | 0.0890 | 0.3575 | -4.3044 | *FXR1* | down |  |
|  |  | 0.8581 | 8.2443 | 1.8233 | 0.0898 | 0.3587 | -4.3118 | *SV2B* | down |  |
|  |  | 1.1841 | 7.6830 | 1.8137 | 0.0914 | 0.3603 | -4.3250 | *MAN1A2* | down |  |
|  |  | 0.5209 | 6.7788 | 1.7909 | 0.0951 | 0.3623 | -4.3562 | *NETO1* | down |  |
|  |  | 0.5210 | 7.5240 | 1.7678 | 0.0990 | 0.3640 | -4.3876 | *GABRA4* | down |  |
|  |  | 0.8501 | 7.7410 | 1.7625 | 0.1000 | 0.3647 | -4.3948 | *SEC62* | down |  |
| Pancreas | GSE20966 | -2.1030 | 31.5930 | -4.7034 | 0.0001 | 0.1312 | 0.9158 | *SRP72* | up | *** |
|  |  | -1.7966 | 21.2083 | -3.9212 | 0.0009 | 0.2582 | -0.5641 | *LPGAT1* | up | *** |
|  |  | -1.6268 | 15.8740 | -3.8190 | 0.0011 | 0.2694 | -0.7596 | *PPM1E* | up | ** |
|  |  | -0.7112 | 17.7733 | -3.5337 | 0.0021 | 0.3588 | -1.3039 | *PSMD7* | up | ** |
|  |  | -0.8665 | 25.7883 | -3.0829 | 0.0059 | 0.5287 | -2.1531 | *DIXDC1* | up | ** |
|  |  | -1.3646 | 22.8521 | -2.6751 | 0.0146 | 0.6431 | -2.8947 | *ANKIB1* | up | * |
|  |  | -1.1911 | 27.2861 | -2.5593 | 0.0187 | 0.6808 | -3.0982 | *RAPH1* | up | * |
|  |  | -1.0558 | 20.4963 | -2.3994 | 0.0263 | 0.7275 | -3.3725 | *SEC24A* | up | * |
|  |  | -2.7149 | 53.1100 | -2.3530 | 0.0290 | 0.7365 | -3.4506 | *ETNK1* | up | * |
|  |  | -0.3678 | 8.9503 | -2.2671 | 0.0347 | 0.7713 | -3.5929 | *RASGEF1B* | up | * |
|  |  | -0.8587 | 28.3246 | -2.2608 | 0.0351 | 0.7715 | -3.6032 | *PAFAH1B1* | up | * |
|  |  | -0.5080 | 11.5181 | -2.1826 | 0.0412 | 0.8129 | -3.7302 | *ZMAT3* | up | * |
|  |  | -0.9662 | 6.1848 | -2.0879 | 0.0499 | 0.8349 | -3.8803 | *SNX16* | up | * |
|  |  | -1.0087 | 34.0960 | -2.0144 | 0.0577 | 0.8518 | -3.9943 | *RORA* | up |  |
|  |  | -0.6513 | 13.0860 | -1.9460 | 0.0659 | 0.8599 | -4.0979 | *KPNA3* | up |  |
|  |  | -0.3435 | 9.7482 | -1.9152 | 0.0699 | 0.8721 | -4.1439 | *MAPK9* | up |  |
|  |  | -1.0548 | 28.8387 | -1.9042 | 0.0714 | 0.8772 | -4.1602 | *ZDHHC21* | up |  |
|  |  | -1.0740 | 12.3727 | -1.8623 | 0.0774 | 0.8884 | -4.2217 | *USP38* | up |  |
|  |  | -0.2583 | 6.4841 | -1.8422 | 0.0804 | 0.8920 | -4.2508 | *ELAVL2* | up |  |
|  |  | -0.5028 | 8.0741 | -1.8184 | 0.0841 | 0.8997 | -4.2851 | *SLC39A10* | up |  |
|  |  | -0.9674 | 10.3460 | -1.7857 | 0.0894 | 0.9000 | -4.3316 | *FAM91A1* | up |  |
|  |  | -0.6300 | 11.9882 | -1.7341 | 0.0984 | 0.9058 | -4.4040 | *STOX2* | up |  |
|  |  | 1.4024 | 17.6955 | 4.2477 | 0.0004 | 0.1957 | 0.0581 | *SV2B* | down | *** |
|  |  | 0.7226 | 22.5161 | 2.5860 | 0.0177 | 0.6794 | -3.0517 | *PTPRF* | down | * |
|  | GSE25724 | -3.5327 | 30.7811 | -6.2021 | 0.0000 | 0.0133 | 2.7003 | *SSR1* | up | *** |
|  |  | -2.7522 | 37.3023 | -5.9364 | 0.0000 | 0.0138 | 2.2997 | *KPNA1* | up | *** |
|  |  | -4.3338 | 22.7513 | -5.0826 | 0.0002 | 0.0150 | 0.9355 | *SH3GLB1* | up | *** |
|  |  | -1.5436 | 11.1280 | -4.9428 | 0.0003 | 0.0153 | 0.7014 | *PPM1A* | up | *** |
|  |  | -2.4587 | 17.2313 | -4.6489 | 0.0004 | 0.0158 | 0.2003 | *MAPRE1* | up | *** |
|  |  | -1.5495 | 7.0757 | -4.5008 | 0.0006 | 0.0169 | -0.0566 | *SHOC2* | up | *** |
|  |  | -4.3287 | 38.3366 | -4.3329 | 0.0008 | 0.0180 | -0.3511 | *SRP72* | up | *** |
|  |  | -3.0844 | 23.1431 | -4.1212 | 0.0012 | 0.0200 | -0.7267 | *PTEN* | up | ** |
|  |  | -1.0672 | 6.9786 | -4.1092 | 0.0012 | 0.0201 | -0.7481 | *PSMD7* | up | ** |
|  |  | -1.0594 | 7.0527 | -4.0697 | 0.0013 | 0.0206 | -0.8188 | *SREK1* | up | ** |
|  |  | -2.3293 | 13.7803 | -3.9475 | 0.0016 | 0.0219 | -1.0380 | *KPNA3* | up | ** |
|  |  | -1.3116 | 6.2091 | -3.9397 | 0.0017 | 0.0219 | -1.0521 | *CDC37L1* | up | ** |
|  |  | -2.3995 | 24.7496 | -3.8149 | 0.0021 | 0.0231 | -1.2773 | *UBE2J1* | up | ** |
|  |  | -1.4085 | 7.7591 | -3.7548 | 0.0024 | 0.0239 | -1.3861 | *DPP8* | up | ** |
|  |  | -0.7757 | 5.9401 | -3.6133 | 0.0031 | 0.0259 | -1.6430 | *CAAP1* | up | ** |
|  |  | -2.2859 | 11.2042 | -3.4930 | 0.0039 | 0.0281 | -1.8619 | *ETNK1* | up | ** |
|  |  | -0.8935 | 3.9025 | -3.4852 | 0.0040 | 0.0283 | -1.8760 | *TBL1XR1* | up | ** |
|  |  | -1.6140 | 15.5607 | -3.4041 | 0.0047 | 0.0302 | -2.0238 | *ARMC8* | up | ** |
|  |  | -0.9737 | 12.9422 | -3.3540 | 0.0051 | 0.0316 | -2.1150 | *SEC24A* | up | ** |
|  |  | -0.8948 | 14.2072 | -3.2228 | 0.0066 | 0.0350 | -2.3539 | *ACTR1A* | up | ** |
|  |  | -1.2012 | 7.1878 | -3.1452 | 0.0077 | 0.0375 | -2.4949 | *UBE3C* | up | ** |
|  |  | -0.6025 | 3.7339 | -3.0236 | 0.0097 | 0.0419 | -2.7151 | *GLCE* | up | ** |
|  |  | -2.8386 | 22.6050 | -2.9776 | 0.0106 | 0.0437 | -2.7982 | *SLC25A36* | up | * |
|  |  | -1.3112 | 12.2877 | -2.9695 | 0.0108 | 0.0439 | -2.8127 | *MAPK9* | up | * |
|  |  | -2.0227 | 31.4301 | -2.9257 | 0.0117 | 0.0455 | -2.8916 | *SON* | up | * |
|  |  | -0.6153 | 8.1650 | -2.9238 | 0.0118 | 0.0455 | -2.8951 | *SETD3* | up | * |
|  |  | -1.0049 | 6.3681 | -2.9222 | 0.0118 | 0.0456 | -2.8980 | *RNF111* | up | * |
|  |  | -1.5464 | 26.5818 | -2.8825 | 0.0128 | 0.0474 | -2.9693 | *DAZAP2* | up | * |
|  |  | -1.9853 | 11.7495 | -2.7708 | 0.0158 | 0.0530 | -3.1688 | *RAP2C* | up | * |
|  |  | -1.0808 | 13.0066 | -2.7495 | 0.0165 | 0.0541 | -3.2066 | *GOLGA4* | up | * |
|  |  | -1.9994 | 26.2712 | -2.6557 | 0.0197 | 0.0591 | -3.3726 | *PAFAH1B1* | up | * |
|  |  | -1.0712 | 5.8563 | -2.6460 | 0.0201 | 0.0598 | -3.3896 | *MEX3C* | up | * |
|  |  | -0.7948 | 13.5041 | -2.5391 | 0.0246 | 0.0676 | -3.5767 | *CCNT2* | up | * |
|  |  | -0.8196 | 12.5483 | -2.5378 | 0.0246 | 0.0677 | -3.5790 | *YTHDC1* | up | * |
|  |  | -1.9414 | 17.0788 | -2.5122 | 0.0259 | 0.0697 | -3.6233 | *ADAM10* | up | * |
|  |  | -0.7121 | 6.7485 | -2.4339 | 0.0300 | 0.0761 | -3.7583 | *SMAD1* | up | * |
|  |  | -0.3698 | 6.9020 | -2.3077 | 0.0380 | 0.0885 | -3.9727 | *NRBP1* | up | * |
|  |  | -0.3993 | 7.5339 | -2.1990 | 0.0464 | 0.1005 | -4.1537 | *SOCS6* | up | * |
|  |  | -1.7548 | 17.6788 | -2.1921 | 0.0470 | 0.1014 | -4.1651 | *PHACTR2* | up | * |
|  |  | -0.6847 | 6.9546 | -2.1460 | 0.0512 | 0.1077 | -4.2406 | *MMD* | up |  |
|  |  | -1.4563 | 15.3263 | -2.0143 | 0.0650 | 0.1264 | -4.4522 | *CHD9* | up |  |
|  |  | -0.2935 | 3.3532 | -1.9964 | 0.0671 | 0.1294 | -4.4804 | *SNX16* | up |  |
|  |  | -1.4745 | 7.0590 | -1.9100 | 0.0783 | 0.1446 | -4.6150 | *LPGAT1* | up |  |
|  |  | -0.4897 | 12.2871 | -1.8686 | 0.0842 | 0.1522 | -4.6782 | *GOLGA1* | up |  |
|  |  | -0.3997 | 4.6289 | -1.8356 | 0.0892 | 0.1588 | -4.7281 | *PLAG1* | up |  |
|  |  | -0.7532 | 13.2798 | -1.8041 | 0.0943 | 0.1650 | -4.7752 | *PTCH1* | up |  |
|  |  | 0.9669 | 8.8044 | 3.8888 | 0.0018 | 0.0222 | -1.1438 | *KCNN3* | down | ** |
|  |  | 0.5449 | 5.2137 | 3.8227 | 0.0021 | 0.0230 | -1.2632 | *SLC24A2* | down | ** |
|  |  | 0.7139 | 6.9101 | 3.4099 | 0.0046 | 0.0301 | -2.0133 | *KCNS1* | down | ** |
|  |  | 2.2611 | 27.5645 | 3.3231 | 0.0054 | 0.0323 | -2.1713 | *AAK1* | down | ** |
|  |  | 1.7718 | 22.4437 | 3.2646 | 0.0061 | 0.0339 | -2.2778 | *LPP* | down | ** |
|  |  | 0.4908 | 3.9363 | 3.2488 | 0.0063 | 0.0343 | -2.3065 | *POU3F3* | down | ** |
|  |  | 0.7193 | 10.3606 | 3.0349 | 0.0095 | 0.0414 | -2.6947 | *PAX5* | down | ** |
|  |  | 0.6503 | 9.9278 | 2.8379 | 0.0139 | 0.0495 | -3.0490 | *SORT1* | down | * |
|  |  | 0.5629 | 4.9623 | 2.6858 | 0.0186 | 0.0574 | -3.3194 | *GABRA4* | down | * |
|  |  | 0.6031 | 20.2898 | 2.6220 | 0.0210 | 0.0616 | -3.4318 | *RNGTT* | down | * |
|  |  | 0.4772 | 6.0229 | 2.4437 | 0.0294 | 0.0753 | -3.7415 | *CD34* | down | * |
|  |  | 0.4335 | 4.5845 | 2.3866 | 0.0328 | 0.0804 | -3.8392 | *ZNF507* | down | * |
|  |  | 1.2186 | 16.7840 | 2.1936 | 0.0469 | 0.1012 | -4.1627 | *CDK19* | down | * |
|  |  | 0.2107 | 3.1385 | 2.1188 | 0.0538 | 0.1111 | -4.2848 | *PDE5A* | down |  |
|  |  | 0.8376 | 19.8199 | 1.9650 | 0.0710 | 0.1347 | -4.5296 | *ENTPD1* | down |  |
|  |  | 0.2452 | 5.2939 | 1.9317 | 0.0753 | 0.1405 | -4.5814 | *DGKH* | down |  |
|  | GSE164416 | -1020.8589 | 1432.6310 | -6.8629 | 0.0000 | 0.0000 | 10.1181 | *RASGEF1B* | up | *** |
|  |  | -113.3947 | 234.4374 | -5.0746 | 0.0000 | 0.0020 | 4.0300 | *EIF5A2* | up | *** |
|  |  | -460.1714 | 491.3356 | -5.0527 | 0.0000 | 0.0020 | 3.9579 | *UNC5D* | up | *** |
|  |  | -162.5155 | 737.4415 | -4.8471 | 0.0000 | 0.0031 | 3.2877 | *CDC37L1* | up | *** |
|  |  | -847.4422 | 2282.4144 | -4.5996 | 0.0000 | 0.0057 | 2.4944 | *MAPRE1* | up | *** |
|  |  | -343.9141 | 607.3444 | -4.2333 | 0.0001 | 0.0120 | 1.3531 | *PPM1E* | up | *** |
|  |  | -116.5913 | 91.4797 | -3.9407 | 0.0002 | 0.0213 | 0.4743 | *GABRA1* | up | *** |
|  |  | -136.2710 | 755.7377 | -3.8854 | 0.0003 | 0.0238 | 0.3120 | *RLIM* | up | *** |
|  |  | -225.4836 | 824.7095 | -3.7698 | 0.0004 | 0.0290 | -0.0233 | *SINHCAF* | up | *** |
|  |  | -141.3252 | 625.9389 | -3.6732 | 0.0005 | 0.0339 | -0.2989 | *DIXDC1* | up | *** |
|  |  | -1316.2003 | 4710.2659 | -3.6176 | 0.0006 | 0.0379 | -0.4556 | *PAFAH1B2* | up | *** |
|  |  | -361.9317 | 2757.7592 | -3.5663 | 0.0008 | 0.0419 | -0.5992 | *HIPK2* | up | *** |
|  |  | -140.7949 | 661.7450 | -3.5610 | 0.0008 | 0.0422 | -0.6139 | *KPNA3* | up | *** |
|  |  | -335.0012 | 1453.3678 | -3.4885 | 0.0010 | 0.0476 | -0.8143 | *SHOC2* | up | *** |
|  |  | -327.9433 | 1517.6827 | -3.3789 | 0.0013 | 0.0571 | -1.1123 | *HIPK3* | up | ** |
|  |  | -254.1599 | 1483.3229 | -3.3418 | 0.0015 | 0.0605 | -1.2118 | *RAB10* | up | ** |
|  |  | -1832.7264 | 5507.3739 | -3.3407 | 0.0015 | 0.0605 | -1.2149 | *ETNK1* | up | ** |
|  |  | -176.2022 | 940.6872 | -3.2817 | 0.0018 | 0.0663 | -1.3715 | *UBE3C* | up | ** |
|  |  | -240.5565 | 1454.8891 | -3.2161 | 0.0022 | 0.0733 | -1.5435 | *WASL* | up | ** |
|  |  | -1745.7561 | 7524.2465 | -3.2089 | 0.0022 | 0.0741 | -1.5623 | *SSR1* | up | ** |
|  |  | -181.5131 | 960.2276 | -3.1114 | 0.0029 | 0.0863 | -1.8135 | *SLC4A8* | up | ** |
|  |  | -177.8598 | 1093.7483 | -3.0903 | 0.0031 | 0.0887 | -1.8671 | *ARMC8* | up | ** |
|  |  | -479.9189 | 2549.5730 | -3.0547 | 0.0035 | 0.0937 | -1.9571 | *SRP72* | up | ** |
|  |  | -86.4443 | 529.6781 | -3.0130 | 0.0039 | 0.0998 | -2.0615 | *FBXO33* | up | ** |
|  |  | -136.2317 | 664.4156 | -2.9840 | 0.0042 | 0.1049 | -2.1335 | *SOCS6* | up | ** |
|  |  | -106.8601 | 558.2964 | -2.9539 | 0.0046 | 0.1101 | -2.2077 | *TAB3* | up | ** |
|  |  | -129.5283 | 966.2408 | -2.6488 | 0.0105 | 0.1625 | -2.9291 | *SEC22C* | up | * |
|  |  | -112.6758 | 616.5704 | -2.5855 | 0.0124 | 0.1758 | -3.0713 | *WNK3* | up | * |
|  |  | -178.4890 | 1178.0328 | -2.5563 | 0.0134 | 0.1819 | -3.1359 | *TMCC1* | up | * |
|  |  | -376.1850 | 1877.2338 | -2.4192 | 0.0189 | 0.2155 | -3.4320 | *PHACTR2* | up | * |
|  |  | -76.6815 | 626.7100 | -2.3303 | 0.0235 | 0.2414 | -3.6169 | *SETD3* | up | * |
|  |  | -54.5601 | 354.9280 | -2.2649 | 0.0275 | 0.2607 | -3.7495 | *ATXN1L* | up | * |
|  |  | -424.6299 | 2527.6667 | -2.2599 | 0.0278 | 0.2621 | -3.7595 | *DAZAP2* | up | * |
|  |  | -50.0276 | 300.1223 | -2.2343 | 0.0295 | 0.2696 | -3.8104 | *CAAP1* | up | * |
|  |  | -133.3554 | 746.9310 | -2.2182 | 0.0307 | 0.2746 | -3.8422 | *RAP2C* | up | * |
|  |  | -71.2651 | 476.9121 | -2.2017 | 0.0319 | 0.2785 | -3.8745 | *CPEB3* | up | * |
|  |  | -186.7544 | 1589.7852 | -2.1661 | 0.0346 | 0.2896 | -3.9437 | *MAP3K13* | up | * |
|  |  | -80.5093 | 473.1102 | -2.1570 | 0.0354 | 0.2919 | -3.9613 | *USP38* | up | * |
|  |  | -1226.5590 | 5967.5763 | -2.1402 | 0.0368 | 0.2965 | -3.9935 | *PTEN* | up | * |
|  |  | -75.8503 | 489.6116 | -2.1256 | 0.0380 | 0.3008 | -4.0214 | *FRS2* | up | * |
|  |  | -83.3393 | 747.2498 | -2.0603 | 0.0441 | 0.3220 | -4.1436 | *XPR1* | up | * |
|  |  | -87.1454 | 690.6332 | -2.0408 | 0.0461 | 0.3269 | -4.1797 | *FAM91A1* | up | * |
|  |  | -181.1261 | 785.7365 | -2.0178 | 0.0485 | 0.3354 | -4.2215 | *SEMA6D* | up | * |
|  |  | -468.8735 | 3249.5364 | -1.8873 | 0.0644 | 0.3793 | -4.4521 | *TBL1XR1* | up |  |
|  |  | -56.8817 | 521.3877 | -1.8514 | 0.0694 | 0.3928 | -4.5132 | *MBTPS2* | up |  |
|  |  | -270.7049 | 3444.3159 | -1.8332 | 0.0721 | 0.3993 | -4.5437 | *TRIP11* | up |  |
|  |  | -29.9868 | 216.2928 | -1.7588 | 0.0841 | 0.4291 | -4.6660 | *ZBTB34* | up |  |
|  |  | -162.6303 | 1072.3502 | -1.7535 | 0.0850 | 0.4309 | -4.6744 | *CNOT6L* | up |  |
|  |  | -119.9900 | 778.9476 | -1.7489 | 0.0859 | 0.4329 | -4.6819 | *SLC39A10* | up |  |
|  |  | -88.3609 | 1603.2542 | -1.6911 | 0.0964 | 0.4566 | -4.7731 | *PAFAH1B1* | up |  |
|  |  | -52.3489 | 474.2028 | -1.6733 | 0.0999 | 0.4652 | -4.8008 | *PTPN4* | up |  |
|  |  | 96.6617 | 621.3261 | 3.5710 | 0.0007 | 0.0415 | -0.5862 | *FOXN3* | down | *** |
|  |  | 119.8171 | 231.9245 | 3.2571 | 0.0019 | 0.0690 | -1.4364 | *KCNN3* | down | ** |
|  |  | 178.7106 | 534.3167 | 3.2131 | 0.0022 | 0.0735 | -1.5513 | *FMN1* | down | ** |
|  |  | 57.4161 | 188.9417 | 3.0745 | 0.0033 | 0.0906 | -1.9072 | *DGKH* | down | ** |
|  |  | 321.9949 | 1655.0760 | 2.9398 | 0.0048 | 0.1119 | -2.2425 | *LPP* | down | ** |
|  |  | 161.2951 | 290.1549 | 2.8167 | 0.0067 | 0.1307 | -2.5393 | *ZEB2* | down | ** |
|  |  | 133.6631 | 196.1953 | 2.7125 | 0.0089 | 0.1489 | -2.7834 | *SLC24A2* | down | ** |
|  |  | 61.3238 | 120.2637 | 2.4321 | 0.0183 | 0.2123 | -3.4047 | *GLIPR1* | down | * |
|  |  | 15.5547 | 23.5138 | 2.0964 | 0.0406 | 0.3109 | -4.0765 | *PAX5* | down | * |
|  |  | 41.1007 | 171.8172 | 2.0589 | 0.0442 | 0.3227 | -4.1464 | *CDK19* | down | * |
|  |  | 5.9840 | 7.9522 | 1.9399 | 0.0575 | 0.3604 | -4.3608 | *TRABD2B* | down |  |
|  |  | 88.0671 | 942.1314 | 1.9309 | 0.0586 | 0.3625 | -4.3766 | *MECP2* | down |  |
|  |  | 401.8492 | 3767.0867 | 1.8480 | 0.0699 | 0.3943 | -4.5189 | *APLP2* | down |  |
| Retina | GSE102485 | -3.0850 | 4.2060 | -7.4465 | 0.0032 | 0.1687 | -1.1901 | *SREK1* | up | ** |
|  |  | -0.1617 | 0.1720 | -6.4762 | 0.0051 | 0.1880 | -1.7268 | *PHACTR2* | up | ** |
|  |  | -0.5717 | 0.5380 | -5.3488 | 0.0092 | 0.2380 | -2.4556 | *SCN1A* | up | ** |
|  |  | -1.6917 | 1.4600 | -3.7558 | 0.0266 | 0.3480 | -3.7416 | *KIF5A* | up | * |
|  |  | -0.2267 | 0.3260 | -2.8545 | 0.0560 | 0.4799 | -4.6393 | *ZFHX4* | up |  |
|  |  | 0.6517 | 1.5340 | 4.7056 | 0.0137 | 0.2778 | -2.9337 | *TRIP12* | down | * |
|  |  | 1.8783 | 2.7980 | 4.1768 | 0.0195 | 0.3050 | -3.3672 | *CAPRIN1* | down | * |
|  |  | 0.2250 | 0.1600 | 2.8345 | 0.0571 | 0.4855 | -4.6609 | *GLIPR1* | down |  |
|  |  | 5.1250 | 8.3700 | 2.7040 | 0.0643 | 0.5116 | -4.8023 | *APLP2* | down |  |
|  |  | 0.6067 | 1.2560 | 2.3467 | 0.0905 | 0.5944 | -5.2029 | *SORT1* | down |  |
|  | GSE160306 | -0.2276 | 16.5818 | -3.2546 | 0.0023 | 0.4659 | -1.5839 | *MAPRE1* | up | ** |
|  |  | -0.2846 | 15.4651 | -2.7462 | 0.0090 | 0.4834 | -2.5924 | *NRP2* | up | ** |
|  |  | -0.1147 | 17.2195 | -2.3495 | 0.0238 | 0.5173 | -3.3042 | *ACSL4* | up | * |
|  |  | -0.1558 | 17.2143 | -2.2980 | 0.0268 | 0.5206 | -3.3908 | *TET3* | up | * |
|  |  | -0.1475 | 17.7566 | -2.1266 | 0.0396 | 0.5583 | -3.6694 | *RLIM* | up | * |
|  |  | -0.1509 | 15.0053 | -2.0018 | 0.0520 | 0.5622 | -3.8619 | *UBE2V1* | up |  |
|  |  | -0.5431 | 13.8999 | -1.9839 | 0.0541 | 0.5668 | -3.8888 | *ELL2* | up |  |
|  |  | -0.2420 | 30.7414 | -1.8407 | 0.0730 | 0.5920 | -4.0966 | *MAP3K9* | up |  |
|  |  | -0.1731 | 18.6808 | -1.8265 | 0.0752 | 0.5957 | -4.1166 | *ABHD2* | up |  |
|  |  | -0.1132 | 19.4908 | -1.7784 | 0.0828 | 0.6036 | -4.1830 | *UNC80* | up |  |
|  |  | -0.1169 | 17.5755 | -1.7369 | 0.0900 | 0.6148 | -4.2394 | *ZBTB7A* | up |  |
|  |  | -0.2604 | 18.6024 | -1.7331 | 0.0907 | 0.6148 | -4.2445 | *ONECUT2* | up |  |
|  |  | -0.0603 | 18.3899 | -1.6857 | 0.0996 | 0.6163 | -4.3073 | *CPSF6* | up |  |
|  |  | 0.1236 | 15.8560 | 2.0103 | 0.0511 | 0.5622 | -3.8490 | *RNGTT* | down |  |
|  |  | 0.1212 | 16.2949 | 1.9122 | 0.0629 | 0.5760 | -3.9944 | *TRDMT1* | down |  |
|  |  | 0.0647 | 18.0297 | 1.8074 | 0.0781 | 0.5977 | -4.1432 | *SENP6* | down |  |
|  |  | 0.1312 | 15.6998 | 1.8006 | 0.0792 | 0.5977 | -4.1526 | *FAM227A* | down |  |

**Supplementary Table 5.** List of genes observed at least four times in upregulated categories and at least four times in downregulated categories across all datasets.

| **Organ** | **Upregulated** | **Downregulated** |
| --- | --- | --- |
| Foot | *FAT4, SPRED1, RAB30, CMTM4, SEMA3D, TRIP11, WNK3, ZCCHC2, ROCK2, SOBP, PEAK1, EZH1, CPEB3, STOX2, UBN2, INO80D, MDM4, GOLGA1, CACNB4, DMTF1, NTRK2, ZBTB20, ZNF362, CREBRF, PTCH1, SLC25A36, KMT2A, SEMA6D, TP53INP1, GOLGA4, ARMC8, CHIC1, ANKIB1, CCNT2, CAAP1, RICTOR, BCL11B, ELK4, CHD9, PTPN4, FRS2, CDC37L1, WEE1, ZBTB18, ZC3H12C, SETD3, SREK1, SLC1A2* | *DENND1B, SORT1, GABRA4, GLIPR1* |
| Kidney | *GOLGA4, ZBTB20, CREBRF, TRIP11* | *RBPJ, KCNN3* |
| Pancreas | *SRP72, LPGAT1, PSMD7, SEC24A, ETNK1, PAFAH1B1, SNX16, KPNA3, MAPK9, CDC37L1, MAPRE1, SHOC2, UBE3C, SSR1, ARMC8, SOCS6, PHACTR2, SETD3, DAZAP2, CAAP1, RAP2C, PTEN, TBL1XR1, PPM1E, DIXDC1, RASGEF1B, USP38, SLC39A10, FAM91A1* | *KCNN3, DGKH, LPP, SLC24A2, PAX5, CDK19* |
| Retina | - | - |

**Supplementary Table 6.** Synthetic miRNA mimic sequences and product information used for HEK293T transfection.

| **miRNA mimic** | **Sequence** | **miRBase ID** | **Product Name** | **Supplier** |
| --- | --- | --- | --- | --- |
| hsa-miR-144 | GGAUAUCAUCAUAUACUGUAAG | MI0000460 | AccuTarget™ Human miRNA mimics | Bioneer |
| hsa-miR-140 | CAGUGGUUUUACCCUAUGGUAG | MI0000456 |  |  |

**Supplementary Table 7.** Synthetic miRNA mimic sequences and product information used for HEK293T transfection.

| **Gene(human)** | **Direction** | **Primer Sequence (5'-3')** | **Primer length** |
| --- | --- | --- | --- |
| *SREK1* | F | TAG TTC CAG CAG GGA AAG GC | 20 |
|  | R | TCT CCA ACT TCT CCT TCC CAG | 21 |

**Supplementary Table 8.** Primer sequences used for mature miRNA quantification using the Mir-X™ system.

| **miRNA** | **Forward primer** | **Primer length** |
| --- | --- | --- |
| hsa-miR-144-3p | TAC AGT ATA GAT GAT GTA CT | 20 |
| hsa-miR-140-3p | TAC CAC AGG GTA GAA CCA CGG | 21 |
